# Laser‐Induced Co‐Doped FePS_3_ with Massively Phosphorus Sulfur Vacancies Nanosheet for Efficient and Highly Stable Electrocatalytic Oxygen Reaction

**DOI:** 10.1002/advs.202501836

**Published:** 2025-04-04

**Authors:** Ruiqi Xu, Guoshuai Fu, Weimi Ding, Yifan Li, Guowei Yang, Peng Yu, Shuang Li, Pu Liu

**Affiliations:** ^1^ State Key Laboratory of Optoelectronic Materials and Technologies Nanotechnology Research Center Guangzhou Key Laboratory of Flexible Electronic Materials and Wearable Devices School of Materials Science & Engineering Sun Yat‐Sen University Guangzhou Guangdong 510275 P. R. China; ^2^ Nano and Heterogeneous Materials Center School of Materials Science and Engineering Nanjing University of Science and Technology Nanjing 210094 P. R. China

**Keywords:** high stability, laser ablation in liquid, OER, rich defects, two‐dimension FePS_3_

## Abstract

Purposely optimizing material structure to reduce the energy change of the rate‐determining step (RDS) for promoting oxygen evolution reaction (OER) catalytic performance is a major strategy to enhance the energy efficiency of electrocatalytic water splitting. Density functional theory (DFT) simulations indicate that creating a large number of defects on or inside the 2D FePS_3_ is very beneficial for its catalytic reaction of OER, especially when there are more defects, the structural diversity of the surface is more conducive to the adsorption and reaction of intermediates. In particular, when Co‐doped FePS_3_ surfaces produce a large number of S and P defects and expose metallic Fe as active sites, its catalytic performance, especially the catalytic stability, is significantly enhanced. A facile and efficient laser‐ablation‐in‐liquid method is then designed to combine Co with 2D layered crystal FePS_3_. Amazingly, the laser‐induced (Fe_0.53_Co_0.46_)PS_3_ sample exhibits excellent OER performance, with an overpotential at 288 mV and a small Tafel slope of 58.3 mV dec^−1^. Moreover, (Fe_0.53_Co_0.46_)PS_3_ operates stably for 138 h at 10 mA cm^−2^ and 27 h at 100 mA cm^−2^, which shows that the stability of (Fe_0.53_Co_0.46_)PS_3_ far exceeds that of most of OER catalysts of Fe─Co system so far, and the comprehensive OER performance is in the first echelon of transition metal catalyst systems. This work proposes an in‐depth understanding of the structural mechanism design of massive phosphorus sulfur vacancies by laser‐induced manufacturing and will shed new light on promoting the stability of transition metal‐based OER catalysts without any precious alternatives.

## Graphical abstract

DFT‐guided defect engineering in 2D FePS_3_ reveals S/P vacancies Co‐doped and exposed Fe sites synergistically enhance OER performance. Laser‐ablation‐induced (Fe_0.53_Co_0.46_)PS_3_ exhibits excellent OER performance (288 mV overpotential, 58.3 mV dec^−1^ Tafel slope) and outstanding stability (138 h at 10 mA cm^−2^, 27h at 100 mA cm^−2^). This work demonstrates that laser‐induced defect engineering is a powerful strategy for high‐performance, ultra‐stable OER electrocatalysts.

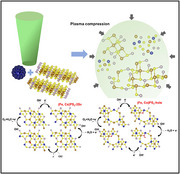

## 1 Introduction

Facing the challenge of climate change and the consumption of fossil fuels, the requirement of renewable and sustainable energy, such as wind energy, solar energy, and tidal energy, is rapidly increasing.^[^ ^1^, ^2^, ^3^ ^]^ Hydrogen (H_2_) has been considered a good alternative and sustainable energy source for fossil fuels due to its high energy density and environmental benignity.^[^ ^4^ ^]^ The most environmentally friendly and ideal production method of hydrogen is using renewable energy to produce hydrogen by electrocatalytic water splitting. Electrocatalytic water splitting can be divided into two half‐reactions: one is oxygen evolution reaction (OER) at the anode, and the other is hydrogen evolution reaction (HER) at the cathode.^[^ ^5^, ^6^ ^]^ Compared to HER, the OER process involves four electron transfers and a variety of intermediate species, and the reaction kinetic sluggishness of OER leads to a higher overpotential to break through the energy barrier.^[^ ^7^ ^]^ Presently, noble metal iridium (Ir) or ruthenium (Ru), especially Ir is the most commonly used OER catalyst in industry, but its scarcity and high cost have brought new challenges to large‐scale industrial applications.^[^ ^8^ ^]^ To achieve large‐scale application in industry and improve the overall efficiency of water electrolysis, in recent years, more and more researchers have begun to pay attention to low‐Ir and non‐Ir OER catalysts, among which the development of transition‐metal‐based OER catalysts has become a major direction.^[^ ^9^ ^]^

2D materials are confirmed to possess excellent optical, electronic, chemical, and mechanical properties.^[^ ^10^ ^]^ The ternary layered material composed of three elements has attracted attention due to its interesting magnetic, and superconductive properties, high chemical diversity, and structural complexity.^[^ ^11^, ^12^ ^]^ Among them, metal phosphorous thiophosphates (MPT) (MPX_3_: M = Co, Fe, Mn and Ni; X = S and Se) are a typical ternary layered material. MPX_3_ belongs to the monoclinic crystal system, and it forms a hexagonal lattice that is stacked along the c‐axis in an ABCABC manner.^[^ ^13^ ^]^ In MPX_3_ crystal, each metal atom is coordinated with six X atoms, and each [P_2_X_6_]^4−^ unit is connected with six M atoms, which are arranged in a honeycomb plane.^[^ ^14^ ^]^ The tunable band structure of MPX_3_ has electron affinity and electronegativity, which makes MPX_3_‐based catalysts become promising in OER and HER.^[^ ^15^, ^16^ ^]^ In comparison to bulk materials, nanosheet materials have a higher specific surface area and more explored active sites. The structural characteristics of nanosheets allow them to design the electronic structure, increase electron transfer, optimize the adsorption of intermediates, and activate the catalytic properties of different elements, like transition metals (Fe, Co, Ni, Mn), providing a new idea for improving the activity of OER and HER catalysts.^[^ ^17^, ^18^, ^19^, ^20^, ^21^, ^22^ ^]^ For example, FeOOH nanorods@NiOOH nanosheets display outstanding OER and HER performance.^[^ ^19^ ^]^ NiPS_3_ nanosheet compounded with graphene has been shown to have excellent OER activities as electrocatalysts.^[^ ^15^ ^]^ However, compared to precious metal‐based OER catalysts, the catalytic stability of MPT nanosheet materials remains a huge challenge in the field of catalytic materials.

Laser ablation in liquids (LAL) is a green, efficient, and simple material preparation technology under ambient temperature, which has been proven that be used for efficiently synthesizing high‐performance nanomaterials or fabricating functional nanostructures.^[^ ^23^ ^]^ In the laser ablation process, LAL can generate extremely high temperatures and pressures in the local liquid phase and ablate the solid target into plasma, which expands, bound by the liquid phase, diffuses, and rapidly cools in the liquid phase. Subsequently, a variety of materials with topological and hybrid structures are formed.^[^ ^24^, ^25^ ^]^ Meanwhile, because the LAL‐generated samples cool rapidly in the liquid‐phase medium at ambient temperature, the samples exhibit a large number of defect‐rich sites.^[^ ^26^ ^]^ For example, some research synthesized high‐performance OER electrocatalysts CoOOH, which was modified by oxygen vacancies by LAL.^[^ ^27^ ^]^

Generally speaking, the kinetics of OER is determined by the rate‐determining step (RDS), and RDS is the step that has the maximum difference of Gibbs chemical binding energy between two subsequent adsorbed intermediates during the four consecutive electrochemical reactions.^[^ ^28^ ^]^ According to our density function theory (DFT) calculation (these are described below), it can be concluded that the difference between the free Gibbs energies of intermediate of OOH* and O* for pristine FePS_3_ is relatively high, which hinders the progress of OER reaction. In order to optimize the adsorption structure and increase the OER kinetics, we attempted to create P and S vacancies in the pristine FePS_3_ calculation model and incorporated Co to replace Fe and bonded. The calculation results show that the adsorption structure is improved compared with pristine FePS_3_. Guided by the DFT calculation, we achieved the recombination of Co and FePS_3_ at ambient temperature using LAL technology and generated a large number of phosphorus and sulfur vacancies while maintaining a layered structure. The OER catalytic performance of (Fe,Co)PS_3_ synthesized by LAL is significantly better than the original FePS_3_. This simple and efficient strategy provides a new path for the recombination of different materials and the optimization and regulation of the performance and structure of the catalyst.^[^ ^24^ ^]^ LAL technology is similar to defect engineering, it can modify the structure of FePS_3_ by laser ablation, provides enough energy to make Co atoms embedded in the FePS_3_ layered structure, and generates a large number of defects. (Fe,Co)PS_3_ forms an undulating surface and creates a large number of vacancies defect after laser ablation, which contributes to the exposure of the active sites for the multi‐step OER reaction, thereby regulating the electronic structure, promoting charge transfer, and contributing to the binding and desorption of intermediate species during the reaction. In this work, through DFT calculation, we realized that the FePS_3_ prepared by LAL exhibits superior OER catalytic performance compared to other defect engineering methods. This is mainly because LAL can generate a large number of surface S and P defects in a small area, exposing the internal Fe to the surface as effective catalytic sites.

Thus, in this work, inspired by DFT design, we attempted to use different mass ratios of FePS_3_ and Co for laser recombination and found that (Fe_0.53_Co_0.46_)PS_3_ (mass ratio) had the best catalytic OER performance, which has the best stability in the Fe─Co OER catalyst system so far. Under alkaline conditions, (Fe_0.53_Co_0.46_)PS_3_ shows a low overpotential on platinum‐carbon electrodes (288 mV, 10 mA cm^−2^), a small Tafel slope of 58.3 mV dec^−1^, and a high catalytic stability under constant current test (10 and 100 mA cm^−2^). This work broadens the application scope of LAL technology and provides a new strategy for the preparation of high‐performance OER catalysts without precious metals.

## 2 Results and Discussion

### 2.1 DFT Design and Rational Prediction

Iron thiophosphates (FePS_3_) are a typical low‐cost layered metal phosphorous thiophosphates (MPT) and have garnered enough attention as electrocatalysts for overall water splitting.^[^ ^13^, ^29^, ^30^ ^]^ However, due to the intrinsic electronic structure characteristic of FePS_3_, the sluggish intermediates species adsorption and dissociation kinetics of FePS_3_ in inferior alkaline water electrolysis are far from being satisfactory for practical applications.^[^ ^29^ ^]^ In order to find a strategy to enhance the OER performance of FePS_3_ and tune the electronic structure of FePS_3_, we first used DFT calculations to display the Gibbs free energy of FePS_3_ during the oxygen evolution reaction process. In alkaline solution, the elementary steps of an OER process are as follows:^[^ ^28^, ^31^, ^32^ ^]^

(R1)

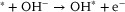

(R2) $${\mathrm{OH}}^{*}+OH^{-}\to O^{*}+H_{2}O+e^{-}$$

(R3) $$O^{*}+OH^{-}\to {\mathrm{OOH}}^{*}+e^{-\;}$$

(R4) $${\mathrm{OOH}}^{*}+OH^{-}\to^{*}+O_{2}+H_{2}O+e^{-}$$

where *, OH*, O*, and OOH* are the native site, the hydroxylated site, the oxidized site, and the oxyhydroxylated site, respectively.

Table S1 (Supporting Information) exhibits the Gibbs free energies change of the calculation models we employed in the OER four‐step reaction. In our calculation, for pristine FePS_3_, considering that the FePS_3_ is composed of FeS_6_ and P_2_S_6_ polyhedra that are linked by edge sharing and Fe atom does not have enough space to adsorb intermediate, we selected nonmetallic P center sites for calculation^[^ ^33^ ^]^ (Figure S1, Supporting Information). For pristine FePS_3_, the rate‐determining step RDS in OER is R3, with a Gibbs free energy change (Δ*G*_3_) of 3.67 eV. This high value of Δ*G*_3_ reflects the weak adsorption of OOH* by FePS_3_ with intact structure, which leads to the inadequate OER performance of pristine FePS_3_, which is consistent with the observations in other articles.^[^ ^13^, ^29^, ^34^ ^]^ In order to enhance the OER activity of FePS_3_, we attempted to introduce one sulfur vacancy in monopoly FePS_3_ (referred as FePS_3_‐Sv). When the adsorption sites take place at the P active sites near the S vacancy (Figure S2, Supporting Information), the Δ*G*_3_ decreased apparently, meanwhile, the intermediate OOH* no longer appeared, decomposed into OH* and O* and adsorbed on S and P, respectively, making R4 the new RDS, with a Gibbs free energy change (Δ*G*_4_) of 3.13 eV. We further created three S vacancies connected to Fe in FePS_3_, referred as FePS_3_‐3 Sv, the adsorption and desorption of intermediates of OER takes place on Fe active sites (Figure S3, Supporting Information), and the Δ*G*_3_ for RDS decreases to 2.75 eV, illustrating the introduction of vacancy in FePS_3_ structure could effectively improve the adsorption of OOH* intermediate, expose more active sites, thus enhancing overall OER efficiency.

**Figure 1:**
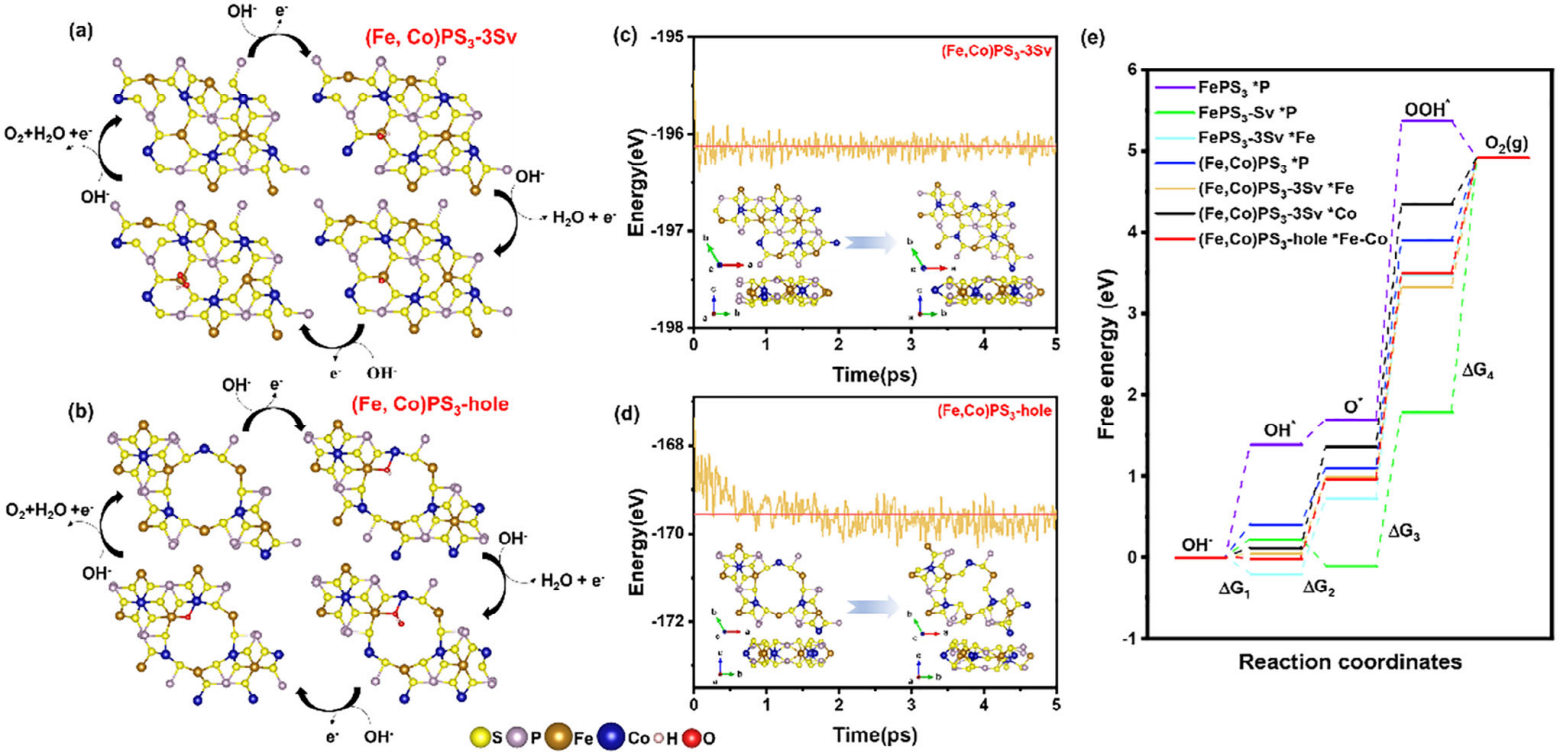
a) the DFT models of OER process of (Fe,Co)PS_3_‐3 Sv with Fe as active sites. b) the DFT models of OER process of (Fe,Co)PS_3_‐hole with Fe─Co bridge as active sites. c,d) Free energy fluctuations with respect to time in AIMD simulations and equilibrium structures of (Fe,Co)PS_3_‐3 Sv c) and (Fe,Co)PS_3_‐Hole d) at 300K e) Free energy changes diagram of OER process for FePS_3_ with different optimized structure.

In addition to considering the introduction of vacancies into FePS_3_ structure, doping cobalt (Co) to replace Fe is also a strategy, and this new structure of Co‐doping is referred as (Fe,Co)PS_3_. First, we calculated the OER Gibbs free energies of (Fe,Co)PS_3_ with P as active sites(Figure S4, Supporting Information). In the case of the same intact structure and metal sites being enclosed by P and S atoms, the RDS of (Fe,Co)PS_3_ is R3 with Δ*G*_3_ of 2.79 eV, which is smaller than the value of pristine FePS_3_ of 3.67 eV, illustrating the doping of Co can also effectively reduce the OER reaction barrier. The preceding part of the text expresses that the introduction of vacancies can improve the OER performance of FePS_3_, so we adopt the same strategy of introducing vacancy defects into (Fe,Co)PS_3_, and attempt to use Fe, Co, or Fe─Co bridge as catalytic sites. For (Fe,Co)PS_3_‐3 Sv with Co as active sites(Figure S5, Supporting Information), the RDS is R3 with Δ*G*_3_ of 2.98 eV, while the active site is Fe, the Δ*G*_3_ decreases to only 2.32 eV, which has the smallest Gibbs free energy change of RDS and means the best‐optimized adsorption structure of FePS_3_. For Fe─Co bridge as active sites, we have tried model of (Fe,Co)PS_3_‐2 Sv, (Fe,Co)PS_3_‐4 Sv, (Fe,Co)PS_3_‐PSv(introduced one P and S vacancy) (Figures S6–S8, Supporting Information), and the ΔG_3_ is 2.76, 3.00, and 3.01 respectively, with limited improvement effect. Furthermore, we introduced hole defect into (Fe,Co)PS_3_ with Fe─Co bridge as active sites, and the Δ*G*_3_ decreased to 2.53 eV. We also attempted to introduce hole defects in (Fe,Co)PS_3_, referred as (Fe,Co)PS_3_‐hole, with Fe─Co bridge as active sites. The RDS is R3 with Δ*G*_3_ of 2.53 eV, which also modified the adsorption structure of OOH* and decreased the energy barrier. For a more intuitive understanding the OER process of introducing vacancies and Cobalt‐doped FePS_3_, **Figure** **1**a,b shows the top views of the OER reaction process of (Fe,Co)PS_3_‐3 Sv(*: Fe) and (Fe,Co)PS_3_‐hole(*: Fe─Co bridge), respectively. Figure 1e exhibits the different free energy changes of adsorptions of surface species (O*, OH*, OOH*) by different reaction sites (*:P, Fe, Co, Fe─Co bridge) on the FePS_3_ with different modified structures. To evaluate the material feasibility, we carried out AIMD calculation to simulate the behavior of (Fe,Co)PS_3_‐3 Sv and (Fe,Co)PS_3_‐hole at 300K, as shown in Figure 1c,d. For (Fe,Co)PS_3_‐3 Sv (Figure 1c), the free energy fluctuates in a narrow range with time evolution at 300K for a duration of 5 ps, and the equilibrium structure remains intact. For (Fe,Co)PS_3_‐hole (Figure 1d), the free energy with time evolution fluctuates greater than the former. It is obvious that the equilibrium structure of (Fe,Co)PS_3_‐Hole has been restructured, but the crystal structure still remains, indicating that defects induced by the action of laser have various structures. The above results demonstrate that FePS_3_ with different defect structures remains stable in the simulation. The above result proves that the doping of Co and the introduction of S and P vacancies can apparently reduce the energy barrier of OER, modulate the structure of pristine FePS_3,_ and enhance the OER property. Guided by the above DFT calculation, we conducted the following experiments.

### 2.2 Synthesis and Characterization of (Fe_0.53_Co_0.46_)PS_3_ Nanostructure

**Figure 2:**
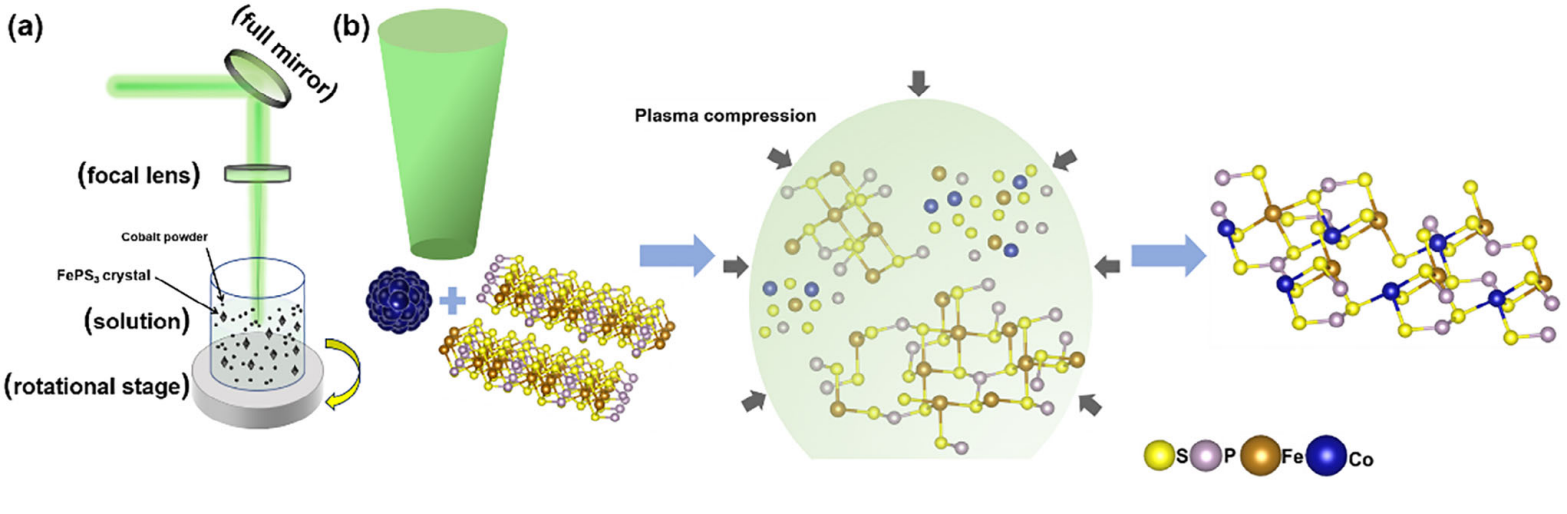
a) LAL synthesis illustration. b) LAL process for the synthesis of (Fe,Co)PS_3_ by LAL.

The high‐quality FePS_3_ crystals were grown using a chemical vapor transport (CVT) method, and then ground the FePS_3_ crystals into powder (see synthesis method in SI), the crystal image of FePS_3_ is shown in Figure S9 (Supporting Information). Different mass ratios of FePS_3_ and Co (FePS_3_:9 mg; Co:1.5, 2, and 2.5 mg) mixed powder were weighed, and then the mixed powder was immersed in the solution of water and isopropanol. After being fully ultrasonicated and dispersed, FePS_3_ and Co mixed powder solution was ablated by high‐energy pulsed laser, and (Fe,Co)PS_3_ (named (Fe_0.66_Co_0.34_)PS_3_, (Fe_0.59_Co_0.41_)PS_3_ and (Fe_0.53_Co_0.46_)PS_3_ according to atomic ratio separately) composites nanomaterials were synthesized. **Figure** **2**a exhibits the fabrication equipment of a room temperature laser‐induced synthesis for (Fe,Co)PS_3_ composites nanomaterial. Figure 2b schematically demonstrates the process of laser ablation to synthesis (Fe,Co)PS_3_ composites. During the process of LAL, under the ablation of pulsed laser, the photons with high energy transferred energy through the coupling of vibrational mode to the material carriers, and the carriers that gain energy collided with the atoms of bulk material and delivered energy into the crystal lattice, creating a high pressure and high‐temperature reaction environment in the local area of the laser action.^[^ ^23^, ^35^ ^]^ Co and FePS_3_ precursors first produced plasma plume under the action of pulsed laser in high‐pressure and high‐temperature condition. Subsequently, the plasma plume expanded rapidly because of the difference of pressure and temperature between plasma and environment, and cavitation bubble collided. Then, due to the constraints of the liquid phase environment, the plasma plume was quickly cooled down and quenched, and formed nanostructure eventually.^[^ ^25^ ^]^ Through selecting appropriate pulsed laser parameters (see laser fabrication method in experimental section), under the action of continuous pulsed laser ablation, 2D FePS_3_ bulk crystal layer was dissociated, the crystal structure of FePS_3_ was partially destroyed, and part of chemical bonds was broken so that a large number of phosphorus and sulfur atoms were able to precipitate from the FePS_3_ crystal, leaving a mass of phosphorus and sulfur vacancies in the FePS_3_ crystal, but still maintaining the basic 2D skeleton. Subsequent structural analysis can demonstrate that (Fe, Co)PS_3_ after being ablated by pulsed laser still maintains the layered structure while producing massive defects of phosphorus and sulfur vacancies.

**Figure 3:**
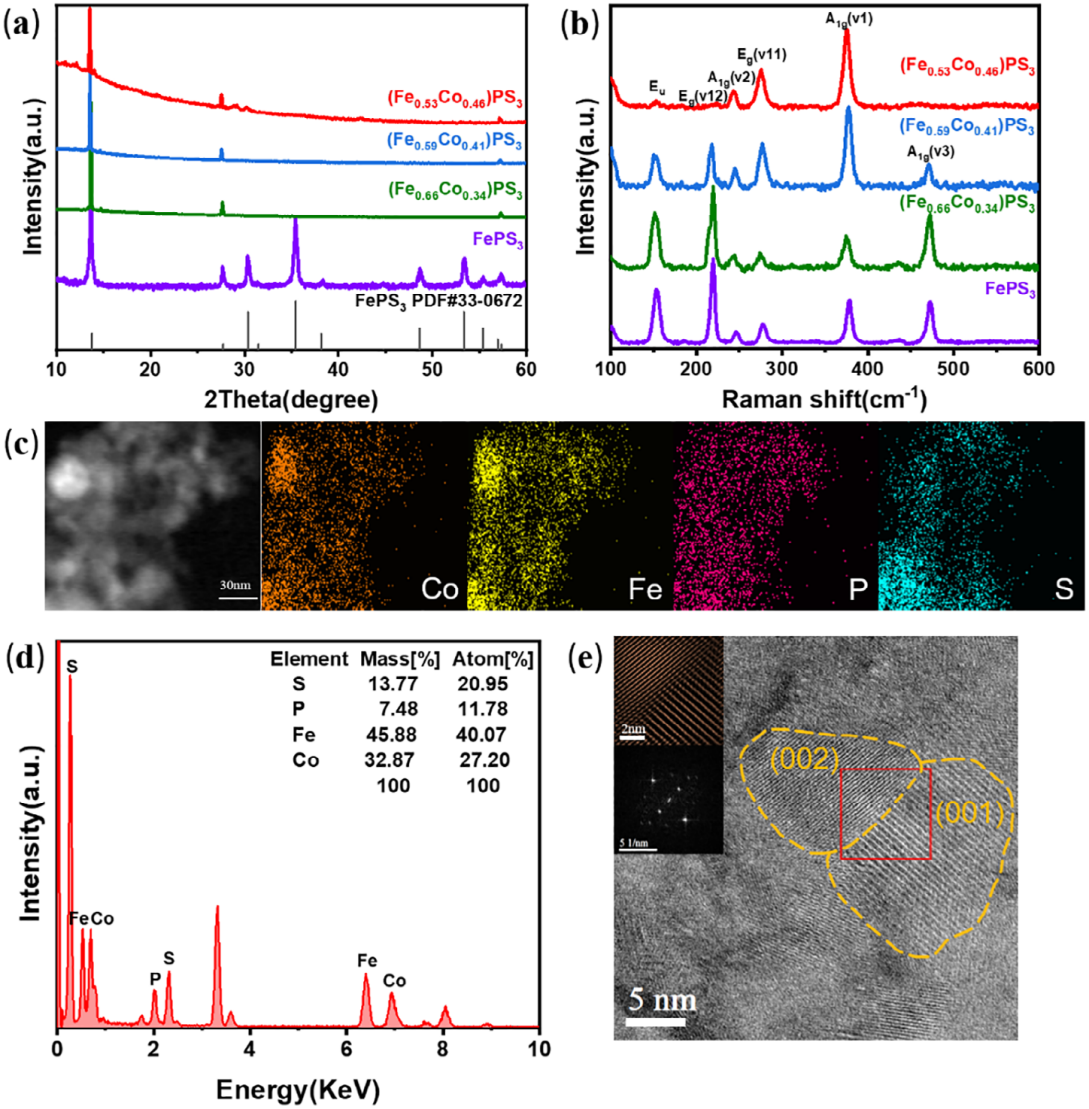
Structure characterization of (Fe,Co)PS_3_. a) XRD patterns of FePS_3_, (Fe_0.66_Co_0.34_)PS_3_, (Fe_0.59_Co_0.41_)PS_3,_ and (Fe_0.53_Co_0.46_)PS_3_, compare with the standard FePS_3_(PDF#33‐0672). b) Raman spectra of FePS_3_, (Fe_0.66_Co_0.34_)PS_3_, (Fe_0.59_Co_0.41_)PS_3,_ and (Fe_0.53_Co_0.46_)PS_3_. c) EDS mapping images of (Fe_0.53_Co_0.46_)PS_3_. d) EDS spectra of (Fe_0.53_Co_0.46_)PS_3_. e) HRTEM image of (001) and (002) crystal plane in (Fe_0.53_Co_0.46_)PS_3_ and selected area electron diffraction (SAED) pattern of (Fe_0.53_Co_0.46_)PS_3_ contains the (001) and (002) zone shown in the inset.

The X‐ray diffraction (XRD) patterns were employed to investigate the structure of FePS_3_ and (Fe,Co)PS_3_ (**Figure** **3**a). As shown in Figure 3a, the diffraction peaks of FePS_3_ used in the experiments fit well with that of the standard FePS_3_ (PDF#30‐0663), proving the high purity of raw FePS_3_ material. The LAL (Fe,Co)PS_3_ samples exhibit major high‐intensity peaks at 2*θ* = 13.54, 27.56, and 57.14°, which correspond to the (001), (002), and (004) planes of FePS_3_, respectively. Meanwhile, we can observe that only the diffraction peaks of partial (00l) planes appear, demonstrating that FePS_3_ crystal structure was destroyed but not completely dissociated into nanoparticles under laser ablation, and still remained a certain crystalline and layered structure. Raman spectrum of FePS_3_ and (Fe,Co)PS_3_ samples at room temperature (293 K) is shown in Figure 3b. The Raman vibration pattern of FePS_3_ can be divided into two modes: one is related to metal cations, with the Raman shift of 0–150 cm^−1^; the other is related to [P_2_S_6_]^4−^, with the Raman shift of 150–600 cm^−1^.^[^ ^36^ ^]^ In Figure 3b, it is obvious that there are similar Raman spectra between FePS_3_ and (Fe,Co)PS_3_. For the Raman peaks of (Fe,Co)PS_3_, the peak at 155 cm^−1^ is related to the E_u_ mode which can be found in all the MPS_3_ compound; the peaks at 220 and 280 cm^−1^ represent the in‐plane E_g_ vibration mode; the peaks at 250(A_1g_ v2) and 380 cm^−1^(A_1g_ v1) are assigned to the out‐of‐plane A_1g_ vibration mode, which is sensitive to alkali‐ion intercalation (A_1g_ v2) and related to the symmetric stretching vibration of the P‐S bond(A_1g_ v1); the peak at 480 cm^−1^ is also associated with the A_1g_(A_1g_ v3)mode, and it mainly involves the P─P bond stretching.^[^ ^34^, ^36^, ^37^, ^38^ ^]^ According to the Raman spectra of (Fe,Co)PS_3_, with the increase of the content of Co ions, the structure of (Fe,Co)PS_3_ remains largely unchanged, and the [P_2_S_6_]^4−^units can be proved to exist, which proves that the layered structure was not completely destroyed under the action of pulsed laser. In addition, for (Fe_0.53_Co_0.46_)PS_3_, the Raman peak at 480 cm^−1^ disappeared, illustrating the disappearance of P─P bond in (Fe_0.53_Co_0.46_)PS_3_, indicating the partial damage of the structure of [P_2_S_6_]^4−^units and the generation of P vacancies. Figure 3c,d displays the energy‐dispersive spectroscopy (EDS) mapping image and EDS spectra of (Fe_0.53_Co_0.46_)PS_3_. The EDS mapping image (Figure 3c) reveals a homogenous distribution of Fe, Co, P, and S elements in the (Fe_0.53_Co_0.46_)PS_3_ nanomaterial. It can be obviously observed that P element is sparser than the other elements, and the elements distribution of Fe and Co in the (Fe_0.53_Co_0.46_)PS_3_ nanostructure matches very well, indicating that Co had been embedded in the FePS_3_ and partly replaced the original Fe sites. In accordance with the EDS spectra(Figure 3d), the atomic ratio of Fe/P/S is 1:0.29:0.52, which is not in agreement with the stoichiometry of FePS_3_, identifying the formation of P and S vacancies.^[^ ^39^ ^]^ Transmission electron microscopy (TEM) images (Figure S9, Supporting Information) show that (Fe_0.53_Co_0.46_)PS_3_ after laser action is layered and irregular shape. High‐resolution TEM (HRTEM) image (Figure 3e) shows two kinds of clear lattice fringe of 0.628 and 0.294 nm, both corresponding to the (001) and (002) planes of FePS_3_ structure, respectively, further confirming a certain degree of crystallinity of (Fe_0.53_Co_0.46_)PS_3_, consistent with the result of XRD. Thus, the XRD, Raman, EDS, and HRTEM analyses confirm that (Fe_0.53_Co_0.46_)PS_3_ is a 2D layered nanomaterial with P and S vacancies.

**Figure 4:**
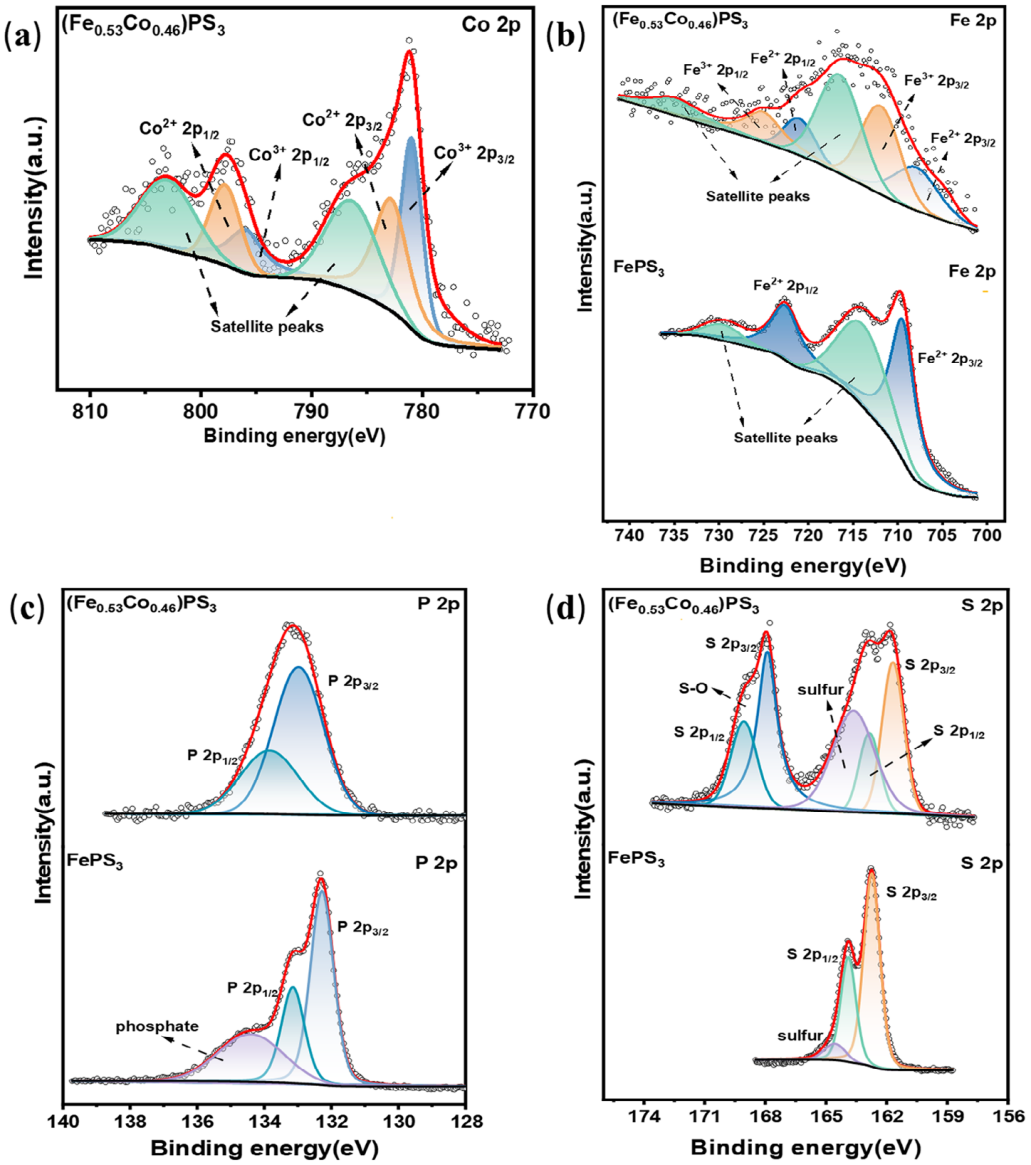
XPS spectra of (Fe_0.53_Co_0.46_)PS_3_ and FePS_3_. a) Co 2p XPS spectra of (Fe_0.53_Co_0.46_)PS_3_. b–d) Fe 2p, P 2p, S 2p XPS spectra of (Fe_0.53_Co_0.46_)PS_3_ and FePS_3_.

X‐ray photoelectron spectroscopy (XPS) was performed to detect the surface chemical state, elemental composition, and electronic structure of original FePS_3_ and (Fe_0.53_Co_0.46_)PS_3_ samples and the resulting peaks were calibrated with C1s spectral peak positions. **Figure** **4**a exhibits the Co 2p XPS spectra of (Fe_0.53_Co_0.46_)PS_3_, which displays two major peaks at 781.28 and 797.78 eV for Co 2p_3/2_ and Co 2p_1/2,_ respectively.^[^ ^40^ ^]^ The 2p_3/2_ and 2p_1/2_ peaks of Co^3+^ are located at 780.96 and 795.95 eV, respectively, and the 2p_3/2_ and 2p_1/2_ peaks of Co^2+^ are observed at 782.82 and 797.81 eV, respectively, indicating that Co exists the mixed valence state of Co^3+^ and Co^2+^ in (Fe_0.53_Co_0.46_)PS_3_. Meanwhile, the spectra exhibit two peaks at 786.35 and 802.99 eV, indicating the satellite peaks of Co.^[^ ^41^ ^]^ Moreover, Co^2+^ is the main form of existence of Co in the (Fe_0.53_Co_0.46_)PS_3_ structure,^[^ ^42^ ^]^ and the relative contents calculated from the fitted area ratios of Co^3+^ and Co^2+^ in the Co 2p spectra are listed in Table S2 (Supporting Information). The XPS spectra of Fe 2p of FePS_3_ and (Fe_0.53_Co_0.46_)PS_3_ are shown in Figure 4b, for (Fe_0.53_Co_0.46_)PS_3_, two major peaks for Fe 2p_3/2_ and Fe 2p_1/2_ locate at 711.2 and 724.6 eV, respectively. Meanwhile, the peaks at 707.91 and 721.01 eV can be attributed to the Fe 2p_3/2_ and Fe 2p_1/2_ for Fe^2+^, and the peaks located at 711.93 and 725.03 eV can be attributed to the Fe 2p_3/2_ and Fe 2p_1/2_ for Fe^3+^, along with two satellite peaks at 716.51 and 734.91 eV for Fe^3+^ 2p_3/2_ and Fe^3+^ 2p_1/2_.^[^ ^43^ ^]^ Compared with FePS_3_, under the action of laser and the embeddedness of Co, we can find that more than half of the Fe atoms are oxidized from Fe^2+^ to Fe^3+^ according to Table S2 (Supporting Information), indicating that the Fe is present in mixed oxidation states (Fe^3+^ and Fe^2+^), similar to Co. In addition, the peak of Fe^2+^ 2p_3/2_ has a negative shift from 709.44 eV (FePS_3_) to 707.91 eV ((Fe_0.53_Co_0.46_)PS_3_), suggesting the presence of electronic interaction between Fe and Co, which is vital for the fast charge transfer in the OER process.^[^ ^40^ ^]^ Furthermore, through partial charge transfer between the cation species of Fe^3+^ and Co^2+^ in (Fe_0.53_Co_0.46_)PS_3_ structure, Fe^3+^ could redistribute the electron density and promote the coordination interaction, which promotes the further oxidation of Co^2+^ and increases OER catalytic activity.^[^ ^44^ ^]^ The P 2p XPS spectra of Figure 4c show that the P 2p_3/2_ and P 2p_1/2_ peaks for (Fe_0.53_Co_0.46_)PS_3_ locate at 132.98 and 133.85 eV, respectively, corresponding to the P‐S bonds in the [P_2_S_6_]^4−^units,^[^ ^45^ ^]^ which can be corroborated with the results of the structural analysis mentioned above, and have a shift to the higher binding energy. Figure 4d displays the S 2p XPS spectra of FePS_3_ and (Fe_0.53_Co_0.46_)PS_3_. For (Fe_0.53_Co_0.46_)PS_3_, the first two peaks at 161.69 and 162.85 eV are respectively related to S 2p_3/2_ and S 2p_1/2_ from transition metal trichalcogenides, moving toward the negative binding energy compared with FePS_3_, and the peak located at 163.64 eV belongs to sulfur. Meanwhile, the last two peaks at 167.90 and 169.06 eV belong to S 2p_3/2_ and S 2p_1/2_, which is attributed to S─O bond, indicating the appearance of oxidized ‐SOx‐ groups in (Fe_0.53_Co_0.46_)PS_3_ samples.^[^ ^46^, ^47^ ^]^

**Figure 5:**
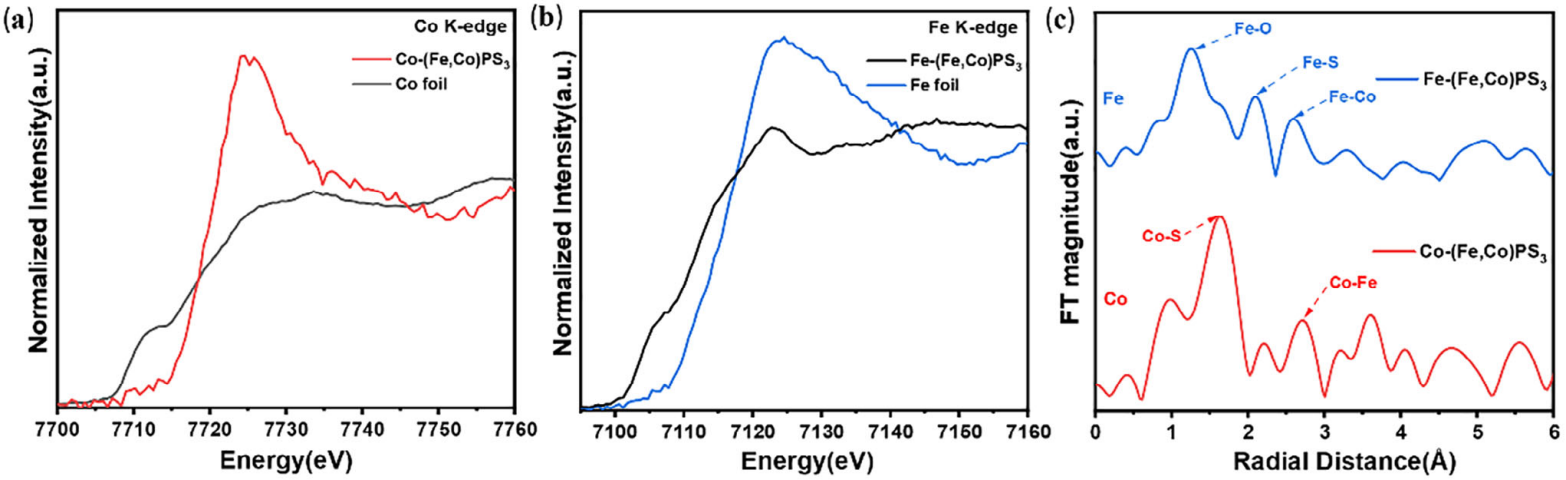
XAS analysis of (Fe,Co)PS_3_ a)Co K‐edge XANES spectra of (Fe,Co)PS_3_ and Co foil b)Fe K‐edge XANES spectra of (Fe,Co)PS_3,_ and Fe foil c)Fourier‐transform FT‐EXAFS spectra of Fe K‐edge and Co K‐edge of (Fe,Co)PS_3._

To further characterize the local structure of (Fe,Co)PS_3_, we performed X‐ray absorption spectroscopy(XAS). The Co K‐edge X‐ray absorption near edge structure (XANES) spectra of (Fe,Co)PS_3_ and Co foil are shown in **Figure** **5**a, which is similar to what reported in the literature for FeCo dual‐atom catalysts,^[^ ^48^, ^49^, ^50^, ^51^ ^]^ indicating Co exists in the form of Co^2+^ and Co^3+^ in (Fe,Co)PS_3_. Figure 5b exhibits the XANES spectra of (Fe,Co)PS_3_ and Fe foil at the Fe K‐edge. Figure 5b suggests that the valence state of Fe in (Fe,Co)PS_3_ mainly presents as Fe^3+^,^[^ ^50^, ^51^ ^]^ illustrating that the valence state of most Fe increases from Fe^2+^ to Fe^3+^ after the laser action and Co doping. The result of XANES spectra of Co K‐edge and Fe K‐edge of (Fe,Co)PS_3_ is consistent with the result of Co 2p and Fe 2p XPS spectra (Figure 4a,b). To explore more structural messages of (Fe,Co)PS_3_, the extended X‐ray absorption fine structure(EXAFS) spectra are shown in Figure S10 (Supporting Information). The Co K‐edge and Fe K‐edge XAFS *k* ^2^ *χ*(*k*) oscillation curves of (Fe,Co)PS_3_ are shown in Figure S11 (Supporting Information). Figure S11 (Supporting Information) shows that there are relatively complete waves in Co K‐edge and Fe K‐edge *k* ^2^ *χ*(*k*) oscillation curves, suggesting that (Fe,Co)PS_3_ after laser ablation still has a certain crystallinity. With the increase of the wavenumber, the waveform is attenuated a bit quickly, illustrating that the formation of defects, like vacancies, makes the disorder of (Fe,Co)PS_3_ increase, which is matched with the above structural analysis. The Fourier‐transform EXAFS spectra of Co K‐edge and Fe K‐edge in (Fe,Co)PS_3_ are shown in Figure 5c. For Co K‐edge, the first peak at 1.63 Å could be assigned to Co‐S bonds,^[^ ^52^, ^53^ ^]^ illustrating that Co had successfully doped into the crystal structure of FePS_3_ and replaced a part of Fe atoms. The third peak at 2.69 Å is connected with Co─Fe bonds, and we can also notice that for Fe K‐edge, the peak at 2.58 Å is consistent with Fe─Co bonds,^[^ ^49^, ^54^ ^]^ demonstrating that Co and Fe formed a synergistic effect. For Fe K‐edge, the peak at 1.26 Å is related to the bonding of Fe^3+^,^[^ ^48^, ^55^ ^]^ and the peak at 2.08 Å originates from the Fe‐S bonds in FePS_3_,^[^ ^32^ ^]^ illustrating that it remained a certain degree FePS_3_ crystal structure.

### 2.3 Electrocatalytic Properties

**Figure 6:**
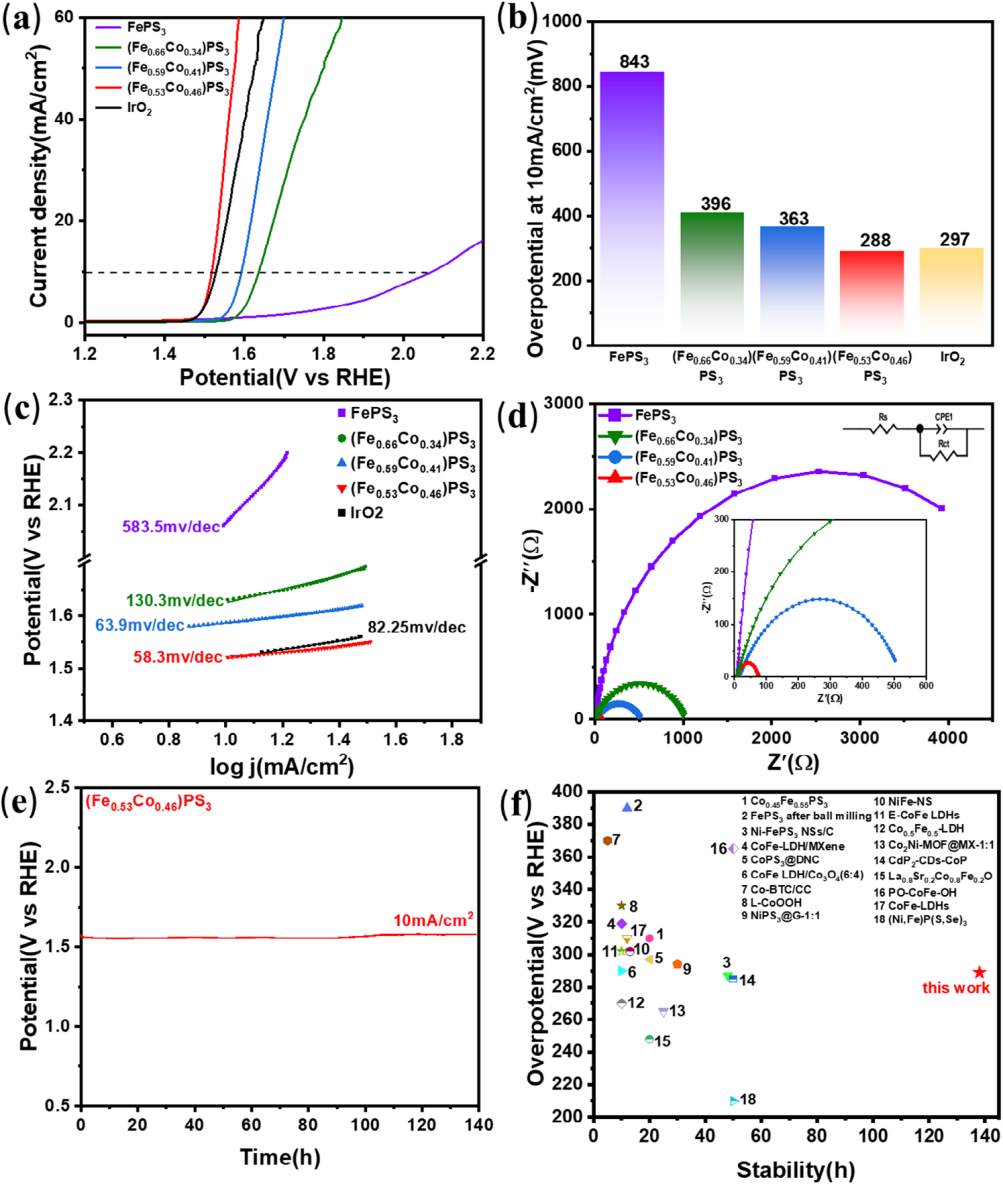
Electrocatalytic OER performance of (Fe,Co)PS_3_. a) LSV curves in 1 m KOH electrolyte at a scan rate of 5mVs^−1^. b) Overpotentials of FePS_3_, (Fe,Co)PS_3_ and IrO_2_ at 10mA cm^−2^. c) Tafel plots. d) EIS of (Fe,Co)PS_3_. e) The 138 h stability test of (Fe_0.53_Co_0.46_)PS_3_ at a constant current density of 10mA cm^−2^. f) Comparison of OER stability and activity of (Fe_0.53_Co_0.46_)PS_3_ catalyst at 10mA cm^−2^.

To investigate the electrocatalytic OER activities of FePS_3_ and (Fe,Co)PS_3_ crystals, we adopt the standard three‐electrode system in the 1.0 m KOH electrolytic solution, which is composed of Hg/HgO electrode as reference electrode, a graphite rod as the counter electrode, and glassy carbon electrode as working electrode. **Figure** **6**a shows the linear sweep voltammetry (LSV) curves of FePS_3_ and (Fe,Co)PS_3_ samples, which indicates that the increase of Co concentration and the generation of P and S vacancies can enhance the current density and promote the overpotential apparently. According to Figure 6b, the pristine FePS_3_ exhibits a poor overpotential of 843 mV at 10mA cm^−2^ for OER. In contrast, the OER performance of (Fe,Co)PS_3_ samples is significantly improved by showing a decreasing overpotential with an increased Co concentration. For (Fe_0.66_Co_0.34_)PS_3_, the overpotential is reduced to 396 mV, and the overpotential of (Fe_0.66_Co_0.34_)PS_3_ is 363 mV. Especially, (Fe_0.53_Co_0.46_)PS_3_ exhibits the best overpotential versus RHE of 288 mV at the current density of 10 mA cm^−2^ on the glassy carbon electrode, which is better than IrO_2_ (297 mV). The Tafel slope is generally used to determine OER kinetics during electrochemical reactions, and the smaller Tafel slope can reflect the higher performance of OER electrocatalysts. The corresponding Tafel slopes also reveal the improved reaction kinetics of (Fe,Co)PS_3_ samples as shown in Figure 6c. The outstanding catalytic reaction kinetics of (Fe_0.53_Co_0.46_)PS_3_ is the lowest Tafel slope value (58.3 mV dec^−1^), which is smaller than that of (Fe_0.59_Co_0.41_)PS_3_ (63.9 mV dec^−1^), (Fe_0.66_Co_0.34_)PS_3_ (130.3 mV dec^−1^), FePS_3_ (583.5 mV dec^−1^), and IrO_2_ (82.25 mV dec^−1^), Hence, (Fe_0.53_Co_0.46_)PS_3_ with a smaller Tafel slope has a significant catalytic ability and a faster OER reaction kinetics with a faster reaction rate for accelerating the rapid oxygen production, indicating that the introduction of vacancy defect and Co element can improve the OER reaction kinetics.

To further investigate the high OER catalytic activity of (Fe,Co)PS_3_, the electrochemical surface areas (ECSA) of these samples were evaluated by the bilayer double layer capacitance (*C*_dl_), which was obtained in a non‐Faradaic region (0.624– 0.824 V vs RHE) through CV test at different scan rates from 20 to 200 mV s^−1^ (Figure S12, Supporting Information). The calculated *C*_dl_ has shown in Figure S13 (Supporting Information) for (Fe_0.53_Co_0.46_)PS_3_, (Fe_0.59_Co_0.41_)PS_3_, (Fe_0.66_Co_0.34_)PS_3_ and FePS_3_ are 1.13, 0.269, 0.258, and 0.201 mF cm^−2^, respectively, indicating the exposure of more active sites of (Fe,Co)PS_3_ due to the action of laser and the increase of Co content. Electrochemical impedance spectroscopy (EIS) can reveal the charge transfer resistance of the catalyst during OER. We use Randle's equivalent circuit model, which consists of the solution resistance (*R*_s_) capacitance (CPE) and the charge‐transfer resistance (*R*_ct_) to fit the Nyquist plots. The obtained Nyquist plot for FePS_3_ and (Fe,Co)PS_3_ electrocatalysts is shown in Figure 6d. Apparently, with the increase of Co content, the charge transfer resistance becomes smaller than the pristine FePS_3_, and (Fe_0.53_Co_0.46_)PS_3_ exhibits the lowest *R*_ct_ (75 Ω), which means the introduction of Co and vacancies can enhance the electroconductivity (detailed *R*_ct_ and *R*_s_ are shown in Table S3a, Supporting Information). Similarly, the laser‐activated (Fe,Co)PS_3_ has a significantly enhanced charge‐transfer capability and thus exhibits much higher OER activity, indicating that increasing the Co content resulted in better charge‐transfer kinetics by lowering the resistance between the electrode and electrolyte. Long‐term stability is an important factor in measuring the performance of OER electrocatalysts. Utilizing the carbon paper (effective electrode area of 0.20 cm^2^) as working electrode with a loading mass of 0.5 mg cm^−2^, we chose (Fe_0.53_Co_0.46_)PS_3_ sample to perform the long‐term stability measurement at a constant current density at 10 mA cm^−2^ (Figure 6e) and 100 mA cm^−2^ (Figure S14, Supporting Information). (Fe_0.53_Co_0.46_)PS_3_ exhibits long‐term stability for 138 h at 10 mA cm^−2^, just attenuating by 1.27%, from 1.558 to 1.578 V versus RHE, which shows no significant activity decay and exhibits excellent stability in alkaline environment, indicating that (Fe_0.53_Co_0.46_)PS_3_ is a high active catalyst that outperforms most reported CoFe and NiFe‐Containing OER catalysts in long‐term operation in alkaline solution (Figure 6f; Table S3b, Supporting Information). Meanwhile, (Fe_0.53_Co_0.46_)PS_3_ also shows a small change under a constant current density at 100 mA cm^−2^ for 27 h, attenuating by 2.91%, from 1.706 to 1.755 V versus RHE. The long‐term stability test of (Fe_0.53_Co_0.46_)PS_3_ indicates that the generation of PS vacancies and the doping of Co after laser action makes (Fe_0.53_Co_0.46_)PS_3_ form a complex structure with multiple active sites, optimizes the electronic structure of catalysts,^[^ ^50^ ^]^ which can effectively improve the stability of (Fe_0.53_Co_0.46_)PS_3_. The comparative OER performances of (Fe_0.53_Co_0.46_)PS_3_ nanomaterial with previously reported similar electrocatalysts are tabulated in Table S3b (Supporting Information).

## 3 Conclusion

In summary, based on exquisite design, the DFT calculations provided us a unique understanding that introducing P and S vacancies defects into FePS_3_, doping Co into to FePS_3_ replace Fe could efficiently decrease the OER reaction energy barrier, optimize the adsorption structure of the intermediate OOH*, and improve the OER performance of FePS_3_. Guided by the result of DFT calculation, we synthesized (Fe,Co)PS_3_ nanosheets by laser ablation in liquid and found the embedding of Co and the generation of massive P and S vacancy defects through the relevant structure test. It was found that (Fe_0.53_Co_0.46_)PS_3_ shows the best OER performance compared to pristine FePS_3_ with an overpotential of 288 mV at 10 mA cm^−2^ and a low Tafel slope of 58.3 mV dec^−1^. Moreover, due to the rich‐vacancy multi‐site structure after laser ablation, (Fe_0.53_Co_0.46_)PS_3_ exhibits excellent durability for 138 h at 10 mA cm^−2^ and 27 h at 100 mA cm^−2^, much higher than most of that of similar Co or Fe─Containing OER catalysts. The experimental results further confirm the theory of DFT calculation that the introduction of vacancy defects and doping Co could enhance the OER performance of FePS_3_. Accordingly, these studies shed new light on the design of high‐performance and high‐stability OER catalysts and provide a new way for further exploring more efficient and economically effective non‐precious catalysts consisting of transition‐metal elements.

## 4 Experimental Section

### Fabrication of (Fe,Co)PS_3_

(Fe,Co)PS_3_ was prepared by facile laser ablation in a liquid process. First, FePS_3_ crystals are ground into powder, and 10 mg of FePS_3_ powder is weighed and mixed with cobalt powder (99.8%, Alfa Aesar). Then, the mixed powder was dispersed in a 50 mL mixture of water and isopropanol (volume ratio of 1:3) in a glass bottle to make suspensions and was ultrasonicated for 30 min at room temperature. Second, the pulsed laser was ablated into the suspensions and focused on the mixed powder FePS_3_ and cobalt, while the entire suspension systems were laser processed under the rotation of an external turnplate. The laser was focused onto the suspension systems by a lens (focus length: 500 mm), and the ultimate (Fe,Co)PS_3_ nanomaterial was obtained under the constant action of laser for 3 h. The Nd:YAG laser parameters were as follows: laser wavelength of 532 nm, pulse width of 10 ns, repetition frequency of 10 Hz, and laser pulse power of 300 mJ.

### Characterization

The XRD of FePS_3_ and (Fe,Co)PS_3_ nanomaterials were obtained by X‐ray diffractometer (SmartLAb) with Cu Kα radiation (*λ* = 1.5418 Å) at 1.2 kW and performed with 2*θ* ranges of 10 to 60°. The Raman spectra were detected by a three‐stage Raman spectrometer (TriVista CRS 557). X‐ray photoelectron spectroscopy (XPS) measurements were taken on an X‐ray photoelectron spectrometer (ESCALAB 250, ThermoFisher), and the binding energies of all elements were calibrated with the binding energy of C1s at 284.80 eV. TEM images, HRTEM images, and EDS elemental mapping were taken on an FEI Tecnai G^2^ F30 transmission electron microscope at an accelerating voltage of 300 kV. The Co K‐edge and Fe K‐edge X‐ray absorption near edge structure (XANES) and the extended X‐ray absorption fine structure (EXAFS) were conducted utilizing a hard X‐ray benchtop absorption fine structure spectrometer (RapidXAFS, Anhui Absorption Spectroscopy Analysis Instrument Co., Ltd.) The obtained XAFS data was processed in Athena (version 0.9.26). The parameters were listed as follows: k weight, 2; R range, 1–3 Å for Fe and Co of (Fe,Co)PS_3_; K range, 3–11.40 Å^−1^ for Co‐(Fe,Co)PS_3_; 3–11.39 Å^−1^ for Fe‐(Fe,Co)PS_3_.

### Electrochemical Measurements

OER measurements were performed on a CHI 760E workstation with a three‐electrode measurement system, which consists of a graphite rod as the counter electrode, Hg/HgO electrode as the reference electrode (filled with 1 m KOH solution), and glassy carbon electrode (Φ = 3 mm) or carbon paper (area of 0.2 cm^2^, used for stability test) as working electrode. The catalyst electrode was fabricated by dropping the ink, and the preparation method of homogeneous ink was as follows. Half of the liquid was taken after the action of laser to dry to obtain (Fe,Co)PS_3_ catalyst, and the rest of the solution was saved tightly sealed in centrifuge tubes. Then the (Fe,Co)PS_3_ catalysts were dispersed in a mixture of **780** uL of deionized water, 200 uL of isopropanol, and 20 uL of 5 wt.% Nafion solution through ultrasonication for 2 h. Finally, the homogeneous ink was dropped onto the glassy carbon electrode and carbon paper to reach a catalyst loading of 0.5 mg cm^−2^, and the working electrodes were dried at room temperature. The OER performance was measured in O_2_‐purged 1 m KOH electrolyte at ambient temperature. The Linear sweep voltammetry (LSV) measurement of (Fe,Co)PS_3_ catalysts was performed with a scan rate of 5 mV s^−1^ in a potential range of 1.2–2.0 V versus RHE. The electrochemical active surface area (ECSA) was evaluated by the bilayer double‐layer capacitance (*C*_dl_). Testing the cyclic voltammetry of (Fe,Co)PS_3_ was at different scan rates (from 20 to 200 mV s^−1^) within the range of 0.624–0.824 V versus RHE. Electrochemical impedance spectroscopy (EIS) was performed in a frequency range of 0.01–100 000 Hz at a bias voltage of 1.524 V versus RHE with a 5 mV amplitude. The stability test used carbon paper (0.2 cm^2^) as a working electrode to record the chronopotentiometry test at a current density of 10 and 100 mA cm^−2^ with a loading mass of 0.5 mg cm^−2^.

## Conflict of Interest

The authors declare no conflict of interest.

## Supporting information



Supporting Information

## Data Availability

The data that support the findings of this study are available from the corresponding author upon reasonable request.
