# Supplementary material for: Laser‐Induced Co‐Doped FePS3 with Massively Phosphorus Sulfur Vacancies Nanosheet for Efficient and Highly Stable Electrocatalytic Oxygen Reaction
Source: Adv Sci (Weinh). 2025 Apr 4;12(26):2501836. doi: 10.1002/advs.202501836 (PMC12244500; doi:10.1002/advs.202501836)
Supplement: Supplementary file 1 — Supporting Information [file ADVS-12-2501836-s001.docx]

**Supporting Information for**

**Laser-induced Co-doped FePS_3_ with massively phosphorus sulfur vacancies nanosheet for efficient and highly stable electrocatalytic oxygen reaction**

Ruiqi Xu,^a,1^ Guoshuai Fu,^a,1^ Weimi Ding,^a^ Yifan Li,^a^ Guowei Yang,^a^ Peng Yu,^a,*^ Shuang Li,^b,*^ Pu Liu^a,*^

*^a^State Key Laboratory of Optoelectronic Materials and Technologies, Nanotechnology Research Center, Guangzhou Key Laboratory of Flexible Electronic Materials and Wearable Devices, School of Materials Science & Engineering,* *Sun Yat-sen University, Guangzhou 510275, Guangdong, P. R. China*

*^b^Nano and Heterogeneous Materials Center, School of Materials Science and Engineering, Nanjing University of Science and Technology, Nanjing 210094, P. R. China*

^1^These authors contributed equally to this work.

*Correspondence: [yupeng9@mail.sysu.edu.cn](mailto:yupeng9@mail.sysu.edu.cn) (P. Yu), [lishuang@njust.edu.cn](mailto:lishuang@njust.edu.cn) (S. Li), [liupu5@mail.sysu.edu.cn](mailto:liupu5@mail.sysu.edu.cn) (P. Liu)

**Synthesis of FePS_3_ crystal:**

The layered FePS_3_ crystals were prepared by chemical vapor transport (CVT) method. The high purity materials of iron powder (99.8%, ZhongNuo Advanced Material (Beijing)Technology), phosphorous powder (98.5%, Aladdin) and sulfur powder (99.5%, Aladdin) in the atomic ratio of Fe:P:S=1:1:3 with a total weight of 1g, were weighted and then sealed into an evacuated 20 cm-long quartz tube under a vacuum of 10^-3^ Torr, which was placed in a two-zone tube furnace. Meanwhile, we used iodine (three to four capsules) as a transport agent and mixed it with the materials powder. The reaction zone was heated to 950 ℃ from room temperature for 30h and held for 7 days, the growth zone was heated to 900 ℃ for 30 h. After that, the quartz tube was naturally cooled down to room temperature. Finally, pure FePS_3_ crystals with black, platelet-like shapes were obtained in the growth zone.

**Computational method**

In this research, all density functional theory (DFT) calculations were carried out using generalized gradient approximation with the Predew-Burke-Ernzerhof (PBE)^[1]^ for exchange-correlation functional as implemented in Vienna ab-Initio Simulation Package (VASP)^[2]^. The k-point grid consisted of 5 × 5 × 1 Monkhorst-Pack points for all calculated models^[3]^. The surfaces were modeled by a periodic slab repeated in super cell of lateral size 2×2 with a vacuum interval of 20 Å between the periodic slabs along the z direction to decouple interactions. During structural relaxation, all tolerances of energy were set to 10^-5^ eV, and a convergence criterion of 0.02 eV/Å was used for the maximum force. The number of plane-wave basis sets was determined by a cutoff energy of 450 eV, and a spin-polarized approach was considered in the modelling. To evaluate the thermal stability of calculation models, the ab initio Molecular dynamics simulations (AIMD) were carried out using a canonical ensemble (NVT) with a Nóse–Hoover thermostat^[4]^ at a temperature of 300K for a duration of 5ps.

The adsorption Gibbs free energy is determined by the expression：

Δ*G*_ads_ = Δ*E*_ads_ + ΔZPE - *T*Δ*S*

where ΔZPE and *T*Δ*S* are the zero-point vibration energy and entropy contribution, respectively. ΔZPE - *T*Δ*S* of the OOH^*^, OH^*^, O^*^ and H^*^ on the surface gives 0.4, 0.35, 0.05, and 0.24 eV, respectively. Note that four proton transfer steps are

| * +OH^-^ → OH* + e^-^ | Δ*G*_1_=Δ*G*_OH*_+1/2Δ*G*_H2_ -Δ*G*_H2O_ -Δ*G*_*_ |
| --- | --- |
| OH* + OH^-^ → O^*^ +H_2_O +e^-^ | Δ*G*_2_=Δ*G*_O*_ + 1/2Δ*G*_H2_ -Δ*G*_OH*_ |
| O^*^ + OH^-^→ OOH*+ e^-^ | Δ*G*_3_=Δ*G*_OOH*_ + 1/2Δ*G*_H2_ -Δ*G*_O*_ -Δ*G*_H2O_ |
| OOH*+ OH^-^ → * +O_2_+H_2_O +e^-^ | Δ*G*_4_=Δ*G*_*_+2Δ*G*_H2O_− 3/2Δ*G*_H2_ + 4.92eV -Δ*G*_OOH*_ |

In this work, the basic calculation models we used are listed as follow: For FePS_3_ (2D structure), Symmetry Group: P312(D3-1); a b c: 5.9469, 5.9469, 35.00; α β γ: 90.00, 90.00, 120.00; adding vacuum in the z-axis in 2D structure FePS_3_. For FePS_3_ (bulk structure), Symmetry Group:C2/M(C2H-3); a b c: 5.852, 10.2652, 6.9942; α β γ: 90.00, 106.6258, 90.00.

**
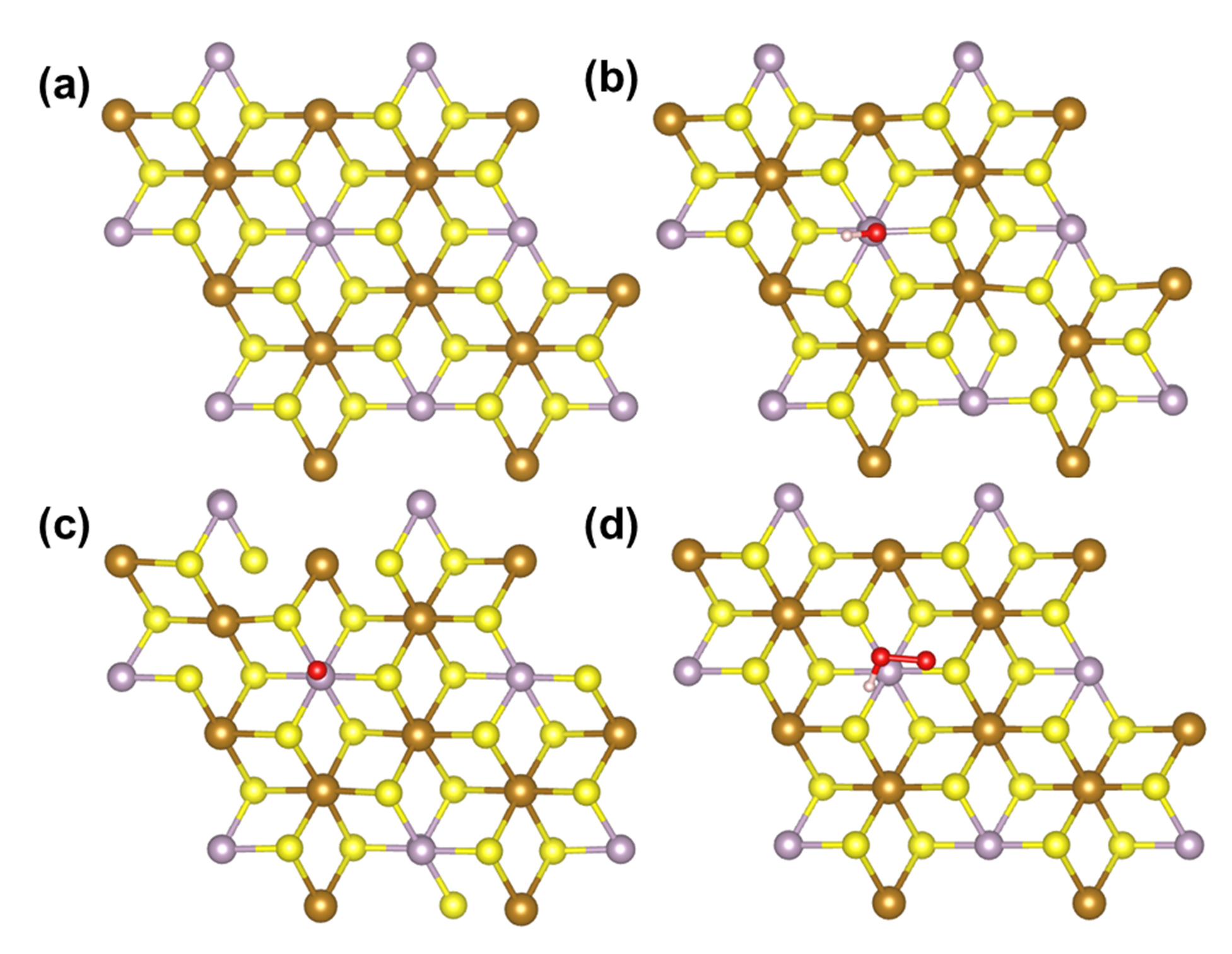
**

**Figure S1.** The optimized structure models of FePS_3_ with the adopted adsorption sites of *(a), OH*(b), O*(c), OOH*(d) on the P sites. Specially, the yellow, purple and brown balls represent S, P and Fe atoms, the red and pink balls represent O and H atoms.

**
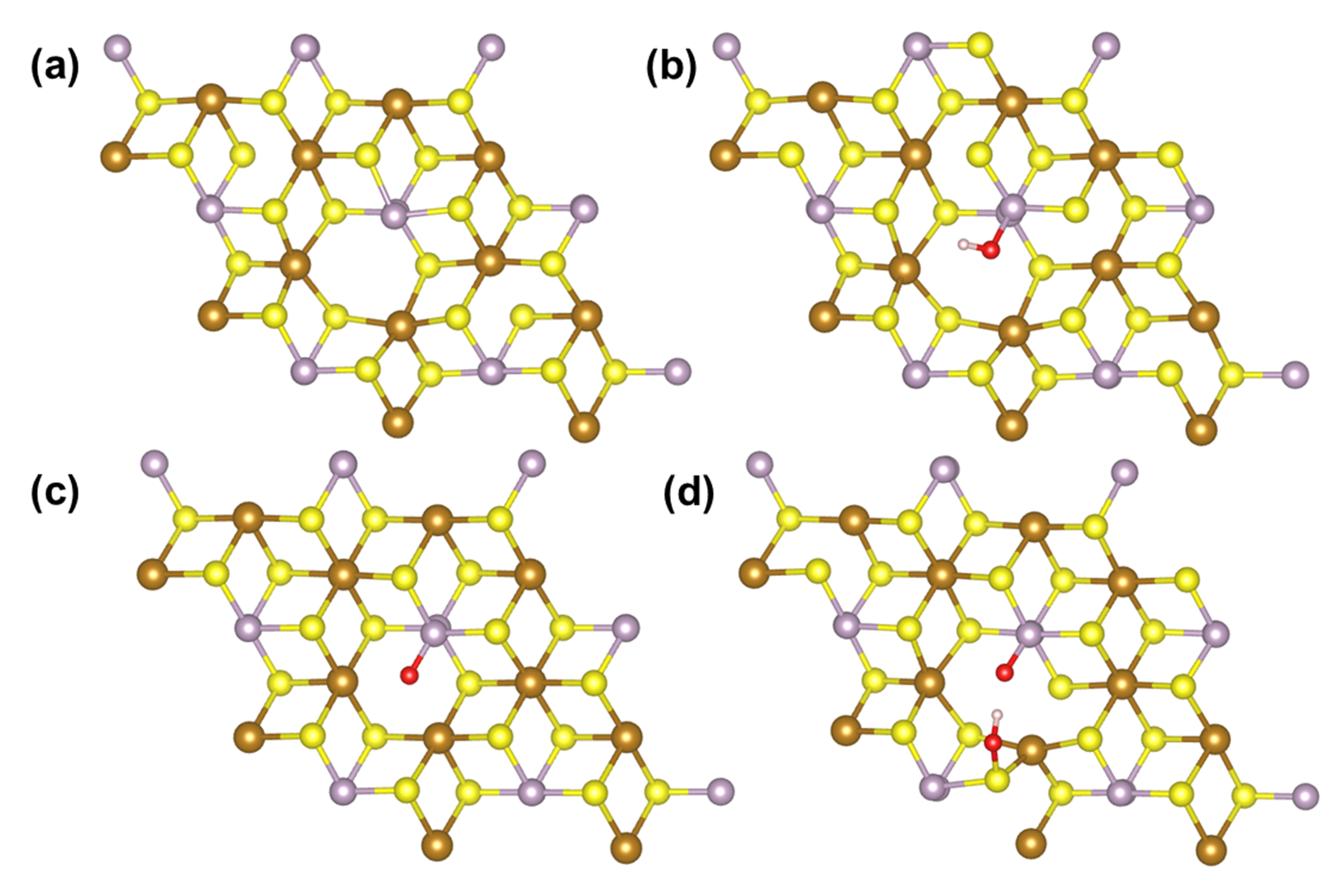
**

**Figure S2.** The optimized structure models of FePS_3_-Sv with the adopted adsorption sites of *(a), OH*(b), O*(c), OOH*(d) on the P sites. Specially, the yellow, purple and brown balls represent S, P and Fe atoms, the red and pink balls represent O and H atoms.

**
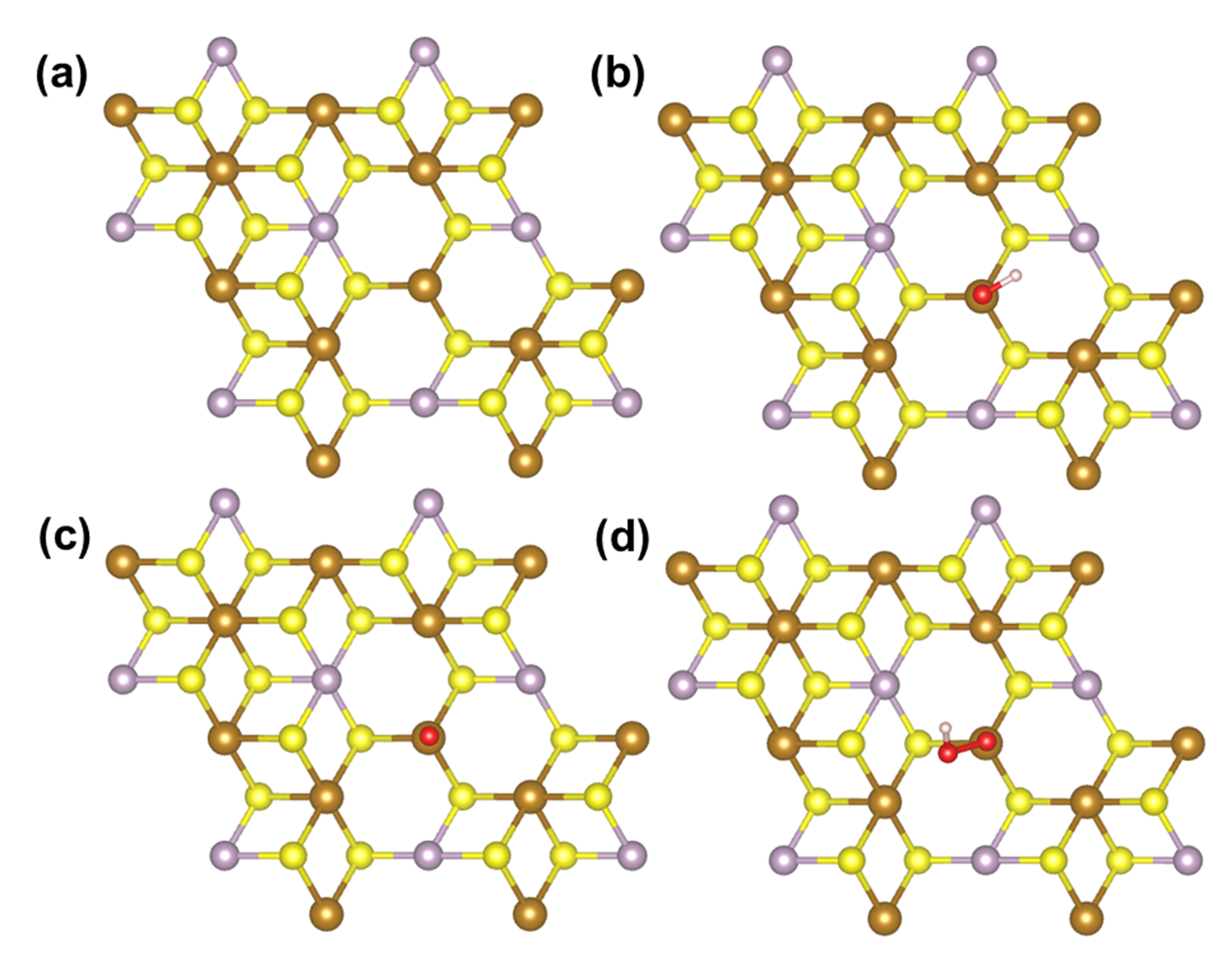
**

**Figure S3.** The optimized structure models of FePS_3_-3Sv with the adopted adsorption sites of *(a), OH*(b), O*(c), OOH*(d) on the Fe sites. Specially, the yellow, purple and brown balls represent S, P and Fe atoms, the red and pink balls represent O and H atoms.

**
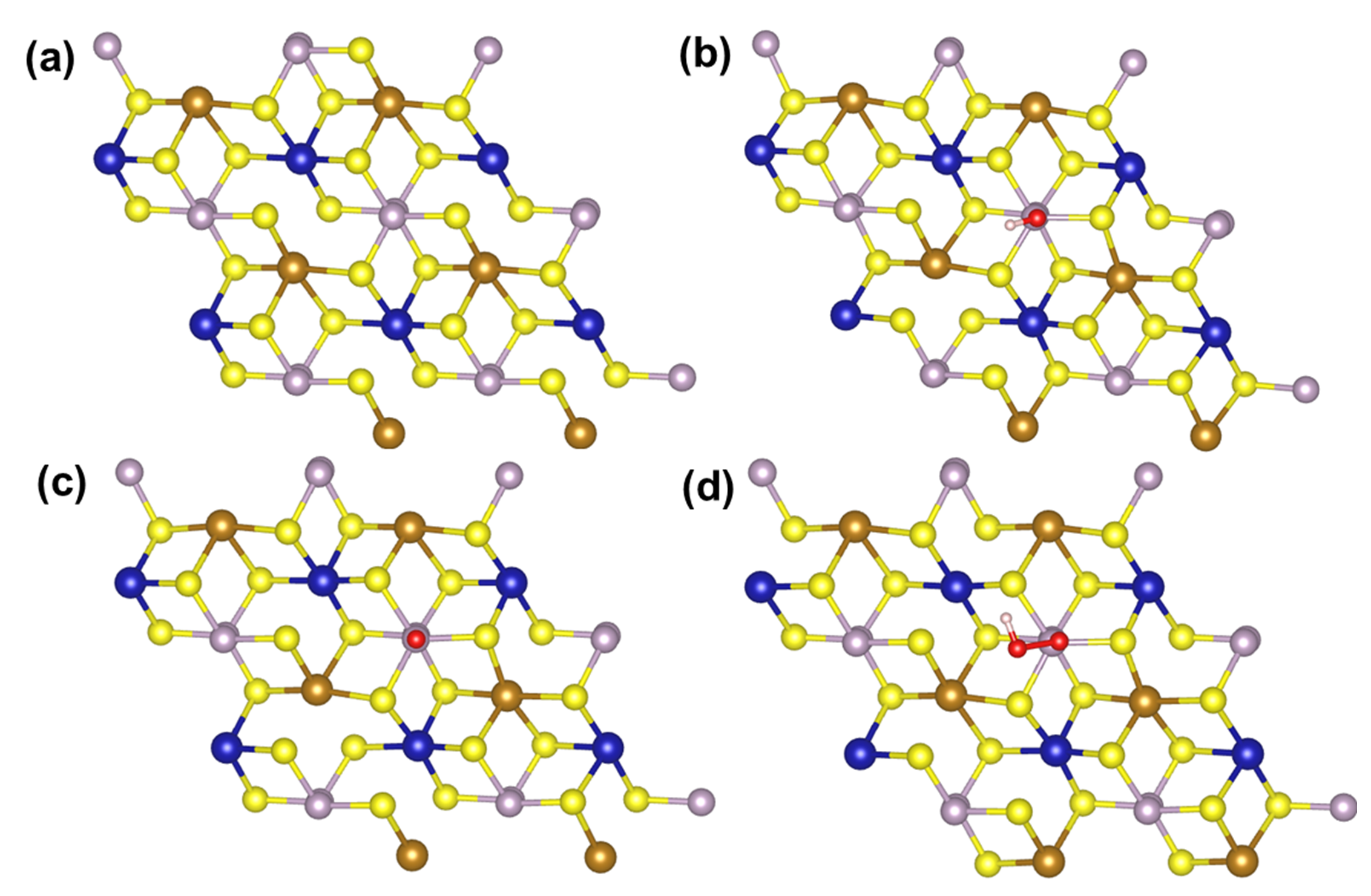
**

**Figure S4.** The optimized structure models of (Fe, Co)PS_3_ with the adopted adsorption sites of *(a), OH*(b), O*(c), OOH*(d) on the P sites. Specially, the yellow, purple balls represent S and P atoms, the brown and blue balls represent Fe and Co atoms, the red and pink balls represent O and H atoms.

**
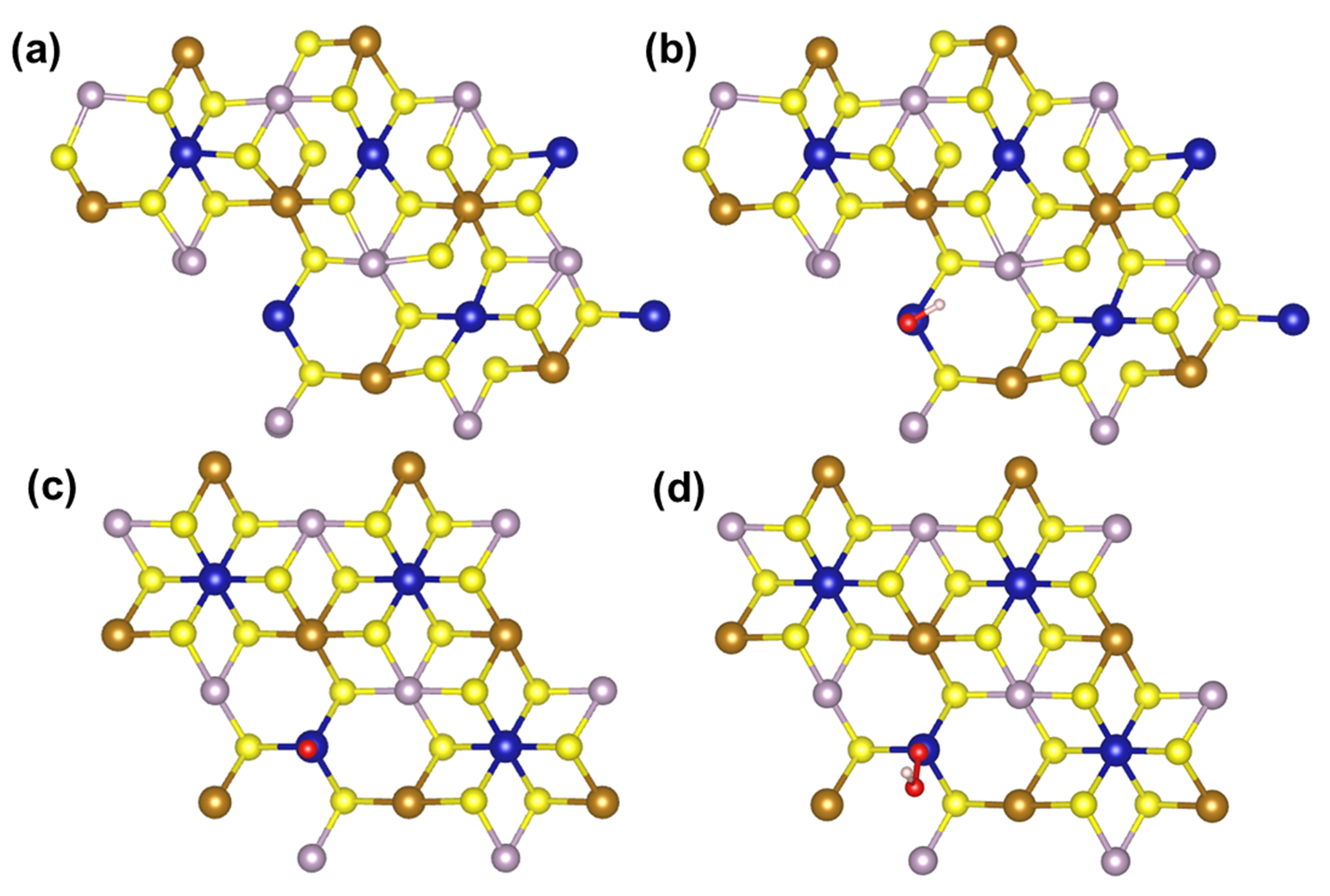
**

**Figure S5.** The optimized structure models of (Fe, Co)PS_3_-3Sv with the adopted adsorption sites of *(a), OH*(b), O*(c), OOH*(d) on the Co sites. Specially, the yellow, purple balls represent S and P atoms, the brown and blue balls represent Fe and Co atoms, the red and pink balls represent O and H atoms.

**
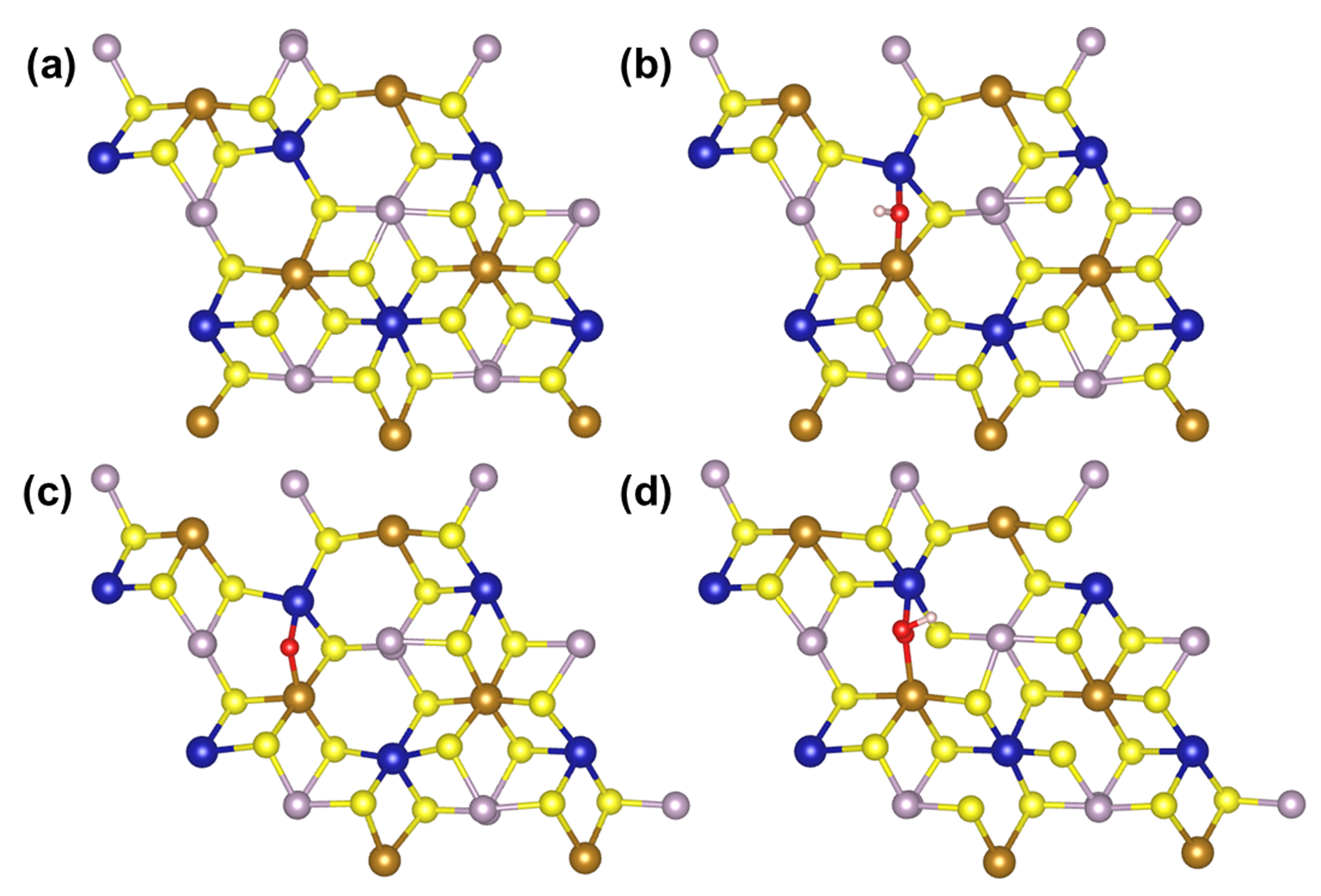
**

**Figure S6.** The optimized structure models of (Fe, Co)PS_3_-2Sv with the adopted adsorption sites of *(a), OH*(b), O*(c), OOH*(d) on the Co-Fe bridge sites. Specially, the yellow, purple balls represent S and P atoms, the brown and blue balls represent Fe and Co atoms, the red and pink balls represent O and H atoms.

**
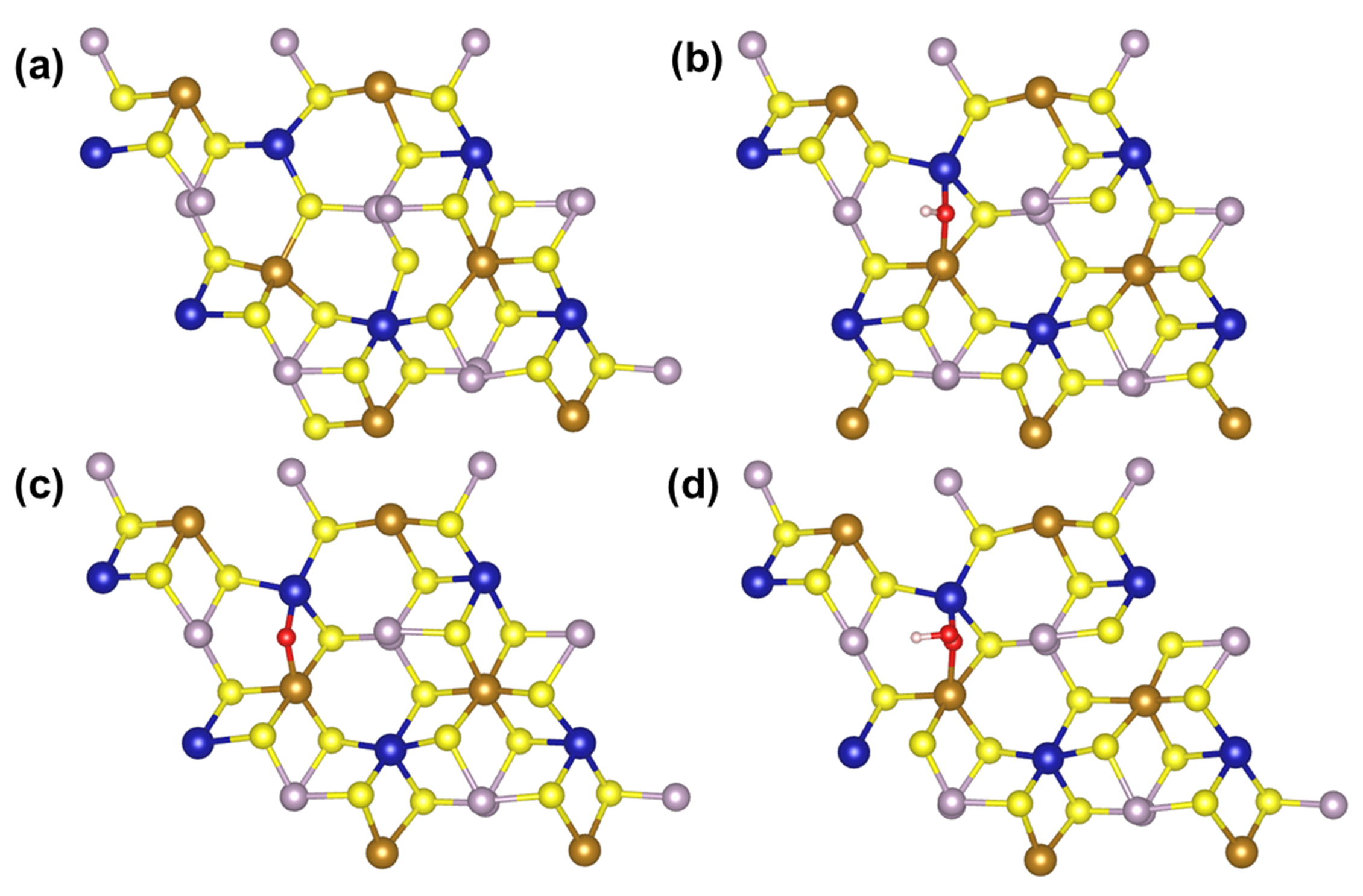
**

**Figure S7.** The optimized structure models of (Fe, Co)PS_3_-4Sv with the adopted adsorption sites of *(a), OH*(b), O*(c), OOH*(d) on the Co-Fe bridge sites. Specially, the yellow, purple balls represent S and P atoms, the brown and blue balls represent Fe and Co atoms, the red and pink balls represent O and H atoms.

**
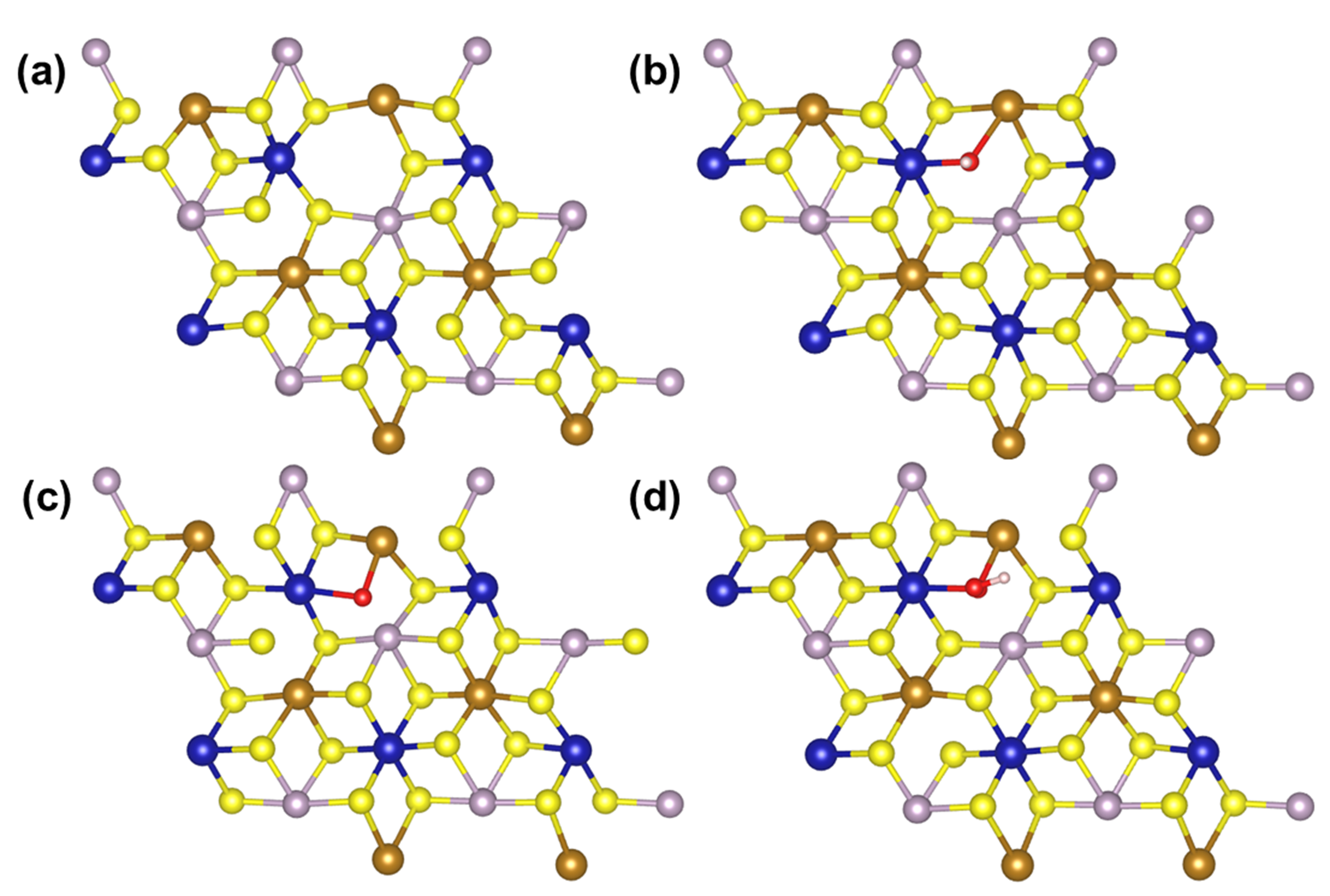
**

**Figure S8.** The optimized structure models of (Fe, Co)PS_3_-PSv with the adopted adsorption sites of *(a), OH*(b), O*(c), OOH*(d) on the Co-Fe bridge sites. Specially, the yellow, purple balls represent S and P atoms, the brown and blue balls represent Fe and Co atoms, the red and pink balls represent O and H atoms.

**
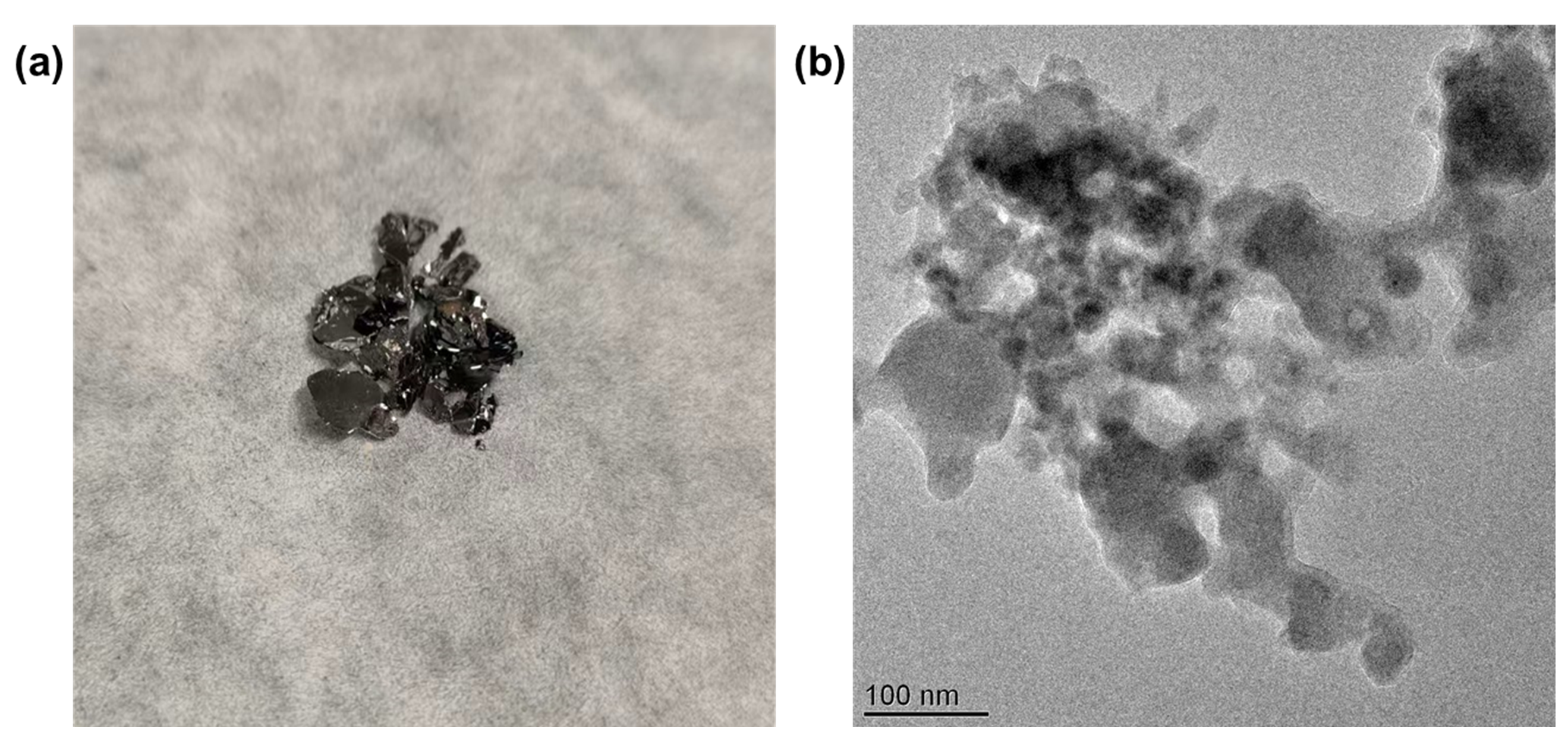
**

**Figure S9.** The photograph of FePS_3_ crystal (a), and TEM image of (Fe_0.53_Co_0.46_)PS_3_ (b).

**
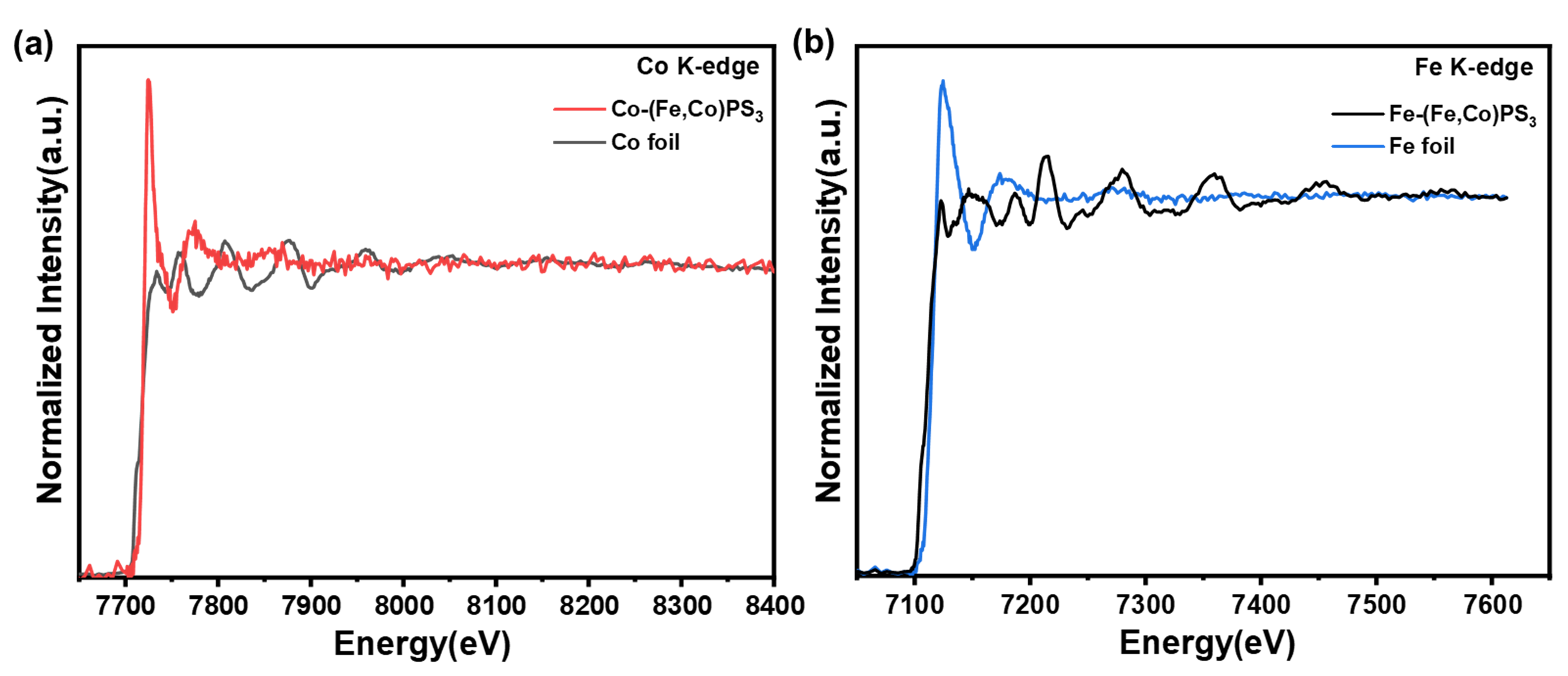
**

**Figure S10.** (a) Co K-edge EXAFS spectra of (Fe,Co)PS_3_ and Co foil. (b) Fe K-edge EXAFS spectra of (Fe,Co)PS_3_ and Fe foil

**
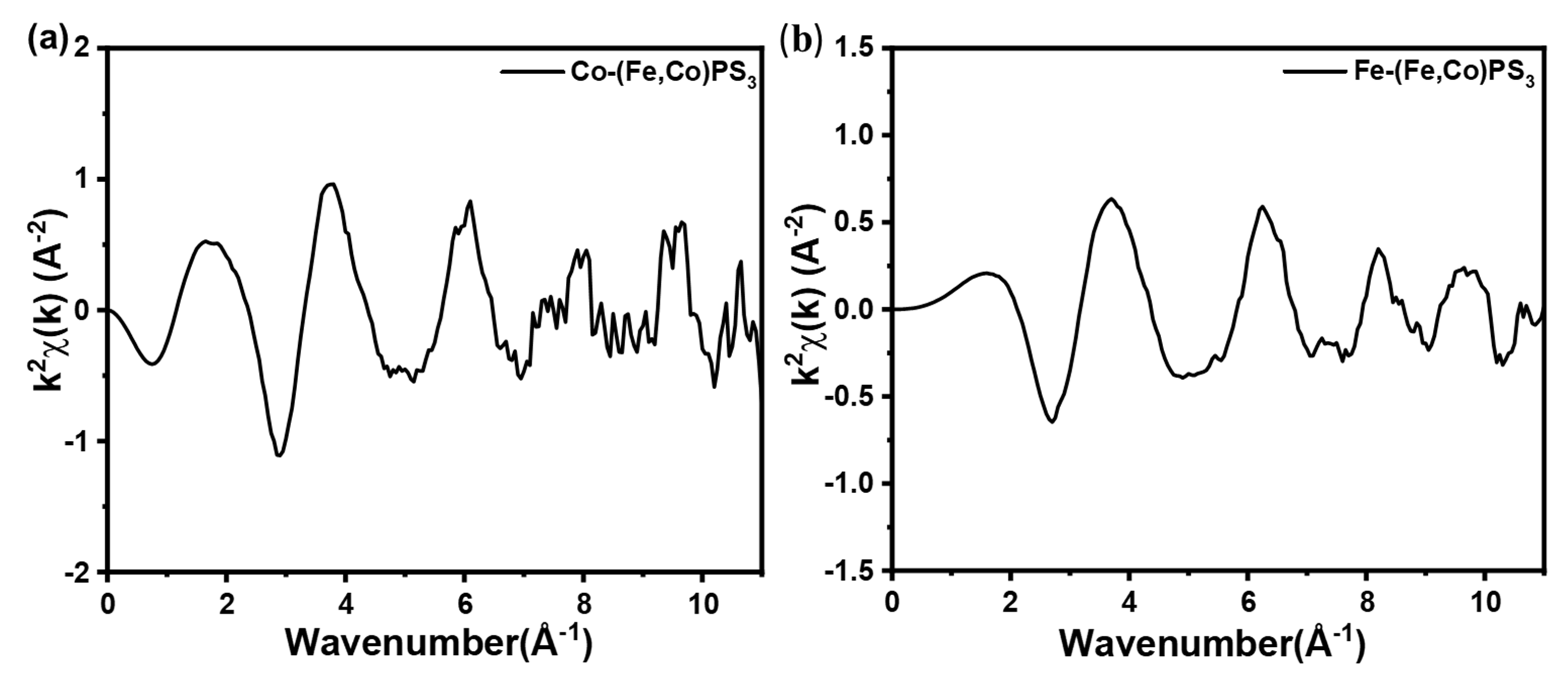
**

**Figure S11.** (a) Co K-edge XAFS *k*^2^*χ*(*k*) oscillation curves of (Fe,Co)PS_3_ (b) Fe K-edge XAFS *k*^2^*χ*(*k*) oscillation curves of (Fe,Co)PS_3_

**
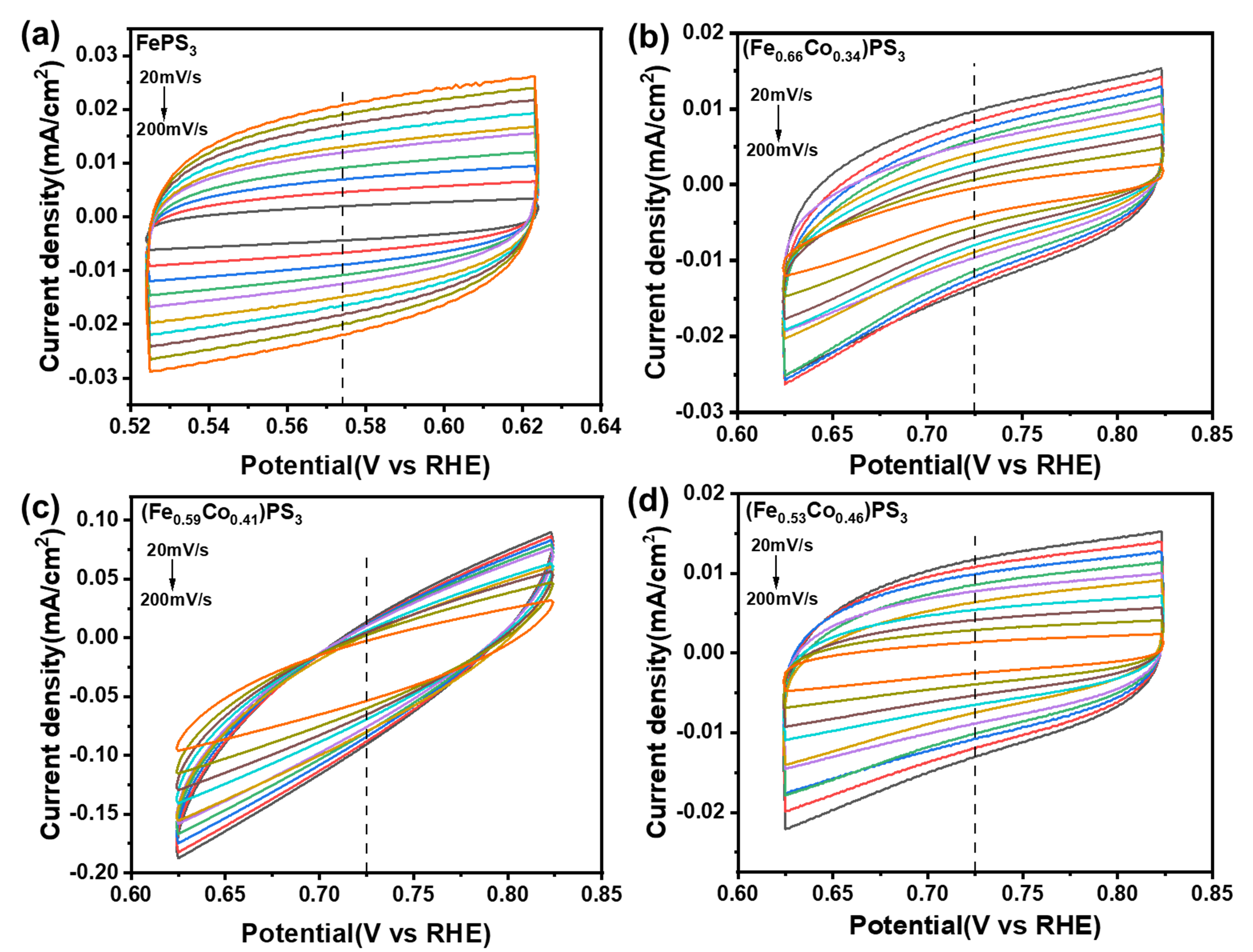
**

**Figure S12.** CV curves at different scan rates from 20 mV/s to 200 mV/s of FePS_3_ (a), (Fe_0.66_Co_0.34_)PS_3_ (b), (Fe_0.59_Co_0.41_)PS_3_ (c), (Fe_0.53_Co_0.46_)PS_3_ (d), respectively.

**
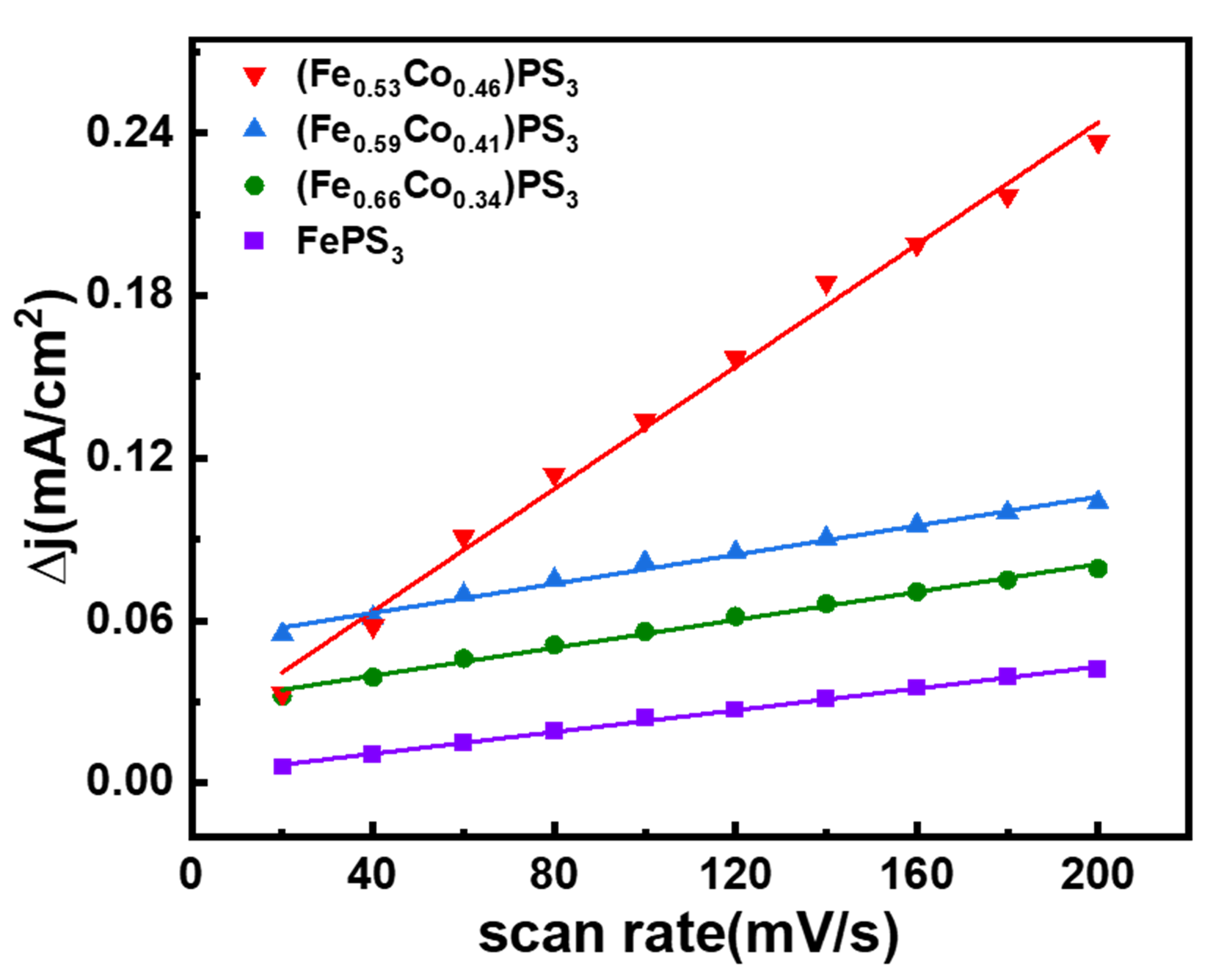
**

**Figure S13.** The double-layer capacitance (C_dl_) curves for original FePS_3_, (Fe_0.66_Co_0.34_)PS_3_, (Fe_0.59_Co_0.41_)PS_3_ and (Fe_0.53_Co_0.46_)PS_3_, respectively.

**
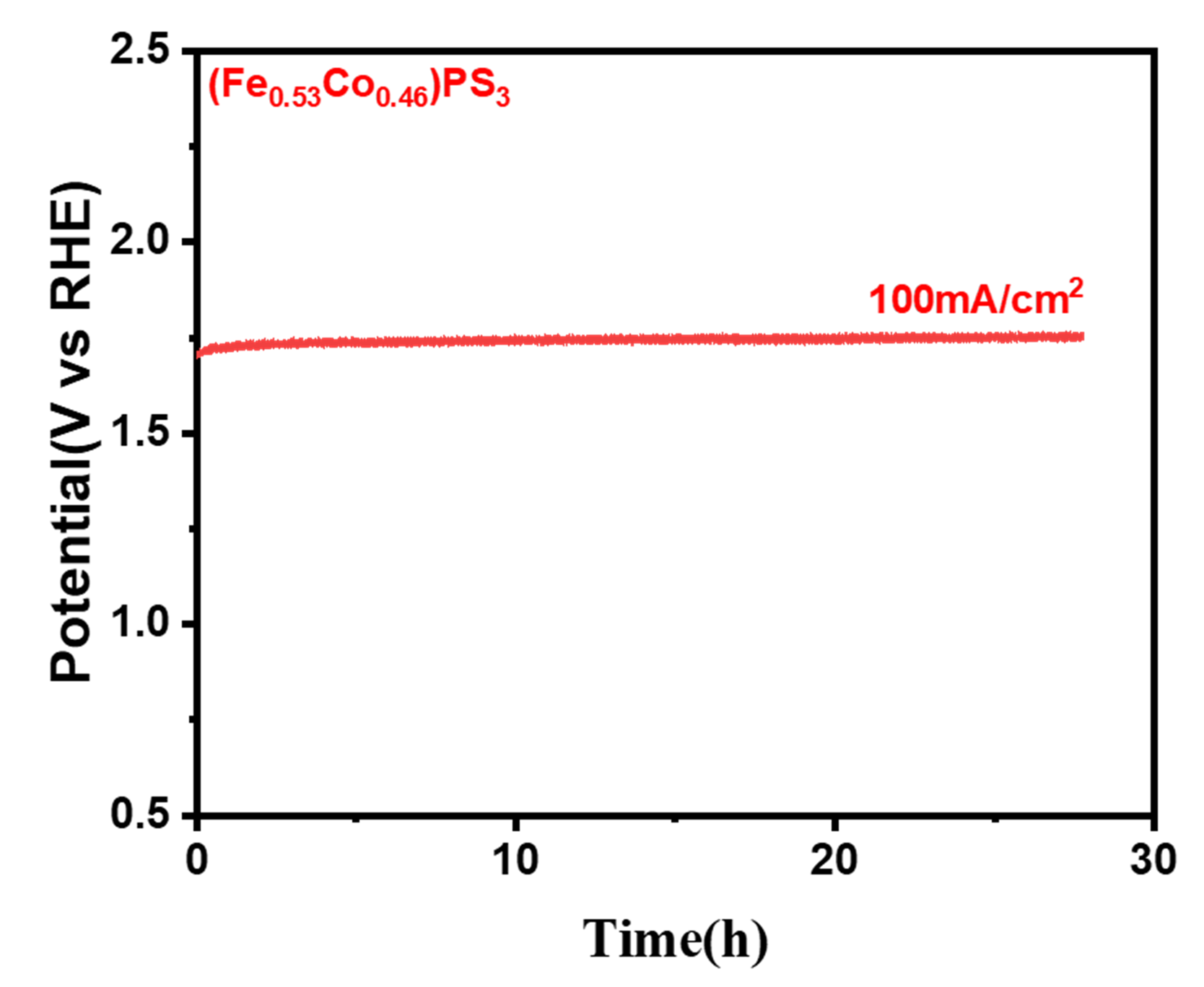
**

**Figure S14.** The long-term stability test curve of (Fe_0.53_Co_0.46_)PS_3_ at a constant current density of 100 mA/cm^2^.

**Table** s1. The Gibbs free energies for the four proton transfer steps of OER reaction for different species of FePS_3_.

|  | Active site | Δ*G*_1_(eV) | Δ*G*_2_(eV) | Δ*G*_3_(eV) | Δ*G*_4_(eV) |
| --- | --- | --- | --- | --- | --- |
| FePS_3_ | P | 1.40 | 0.30 | **3.67** | -0.45 |
| FePS_3_-Sv | P | 0.23 | -0.33 | 1.89 | **3.13** |
| FePS_3_-3Sv | Fe | -0.20 | 0.93 | **2.75** | 1.44 |
| (Fe, Co)PS_3_ | P | 0.41 | 0.70 | **2.79** | 1.02 |
| (Fe, Co)PS_3_-3Sv | Fe | 0.05 | 0.95 | **2.32** | 1.6 |
| (Fe, Co)PS_3_-3Sv | Co | 0.12 | 1.25 | **2.98** | 0.57 |
| (Fe, Co)PS_3_-2Sv | Fe-Co bridge | 0.64 | 1.05 | **2.75** | 0.48 |
| (Fe, Co)PS_3_-4Sv | Fe-Co bridge | -0.19 | 0.59 | **3.00** | 1.51 |
| (Fe, Co)PS_3_-PSv | Fe-Co bridge | -0.33 | 0.33 | **3.01** | 1.91 |
| (Fe, Co)PS_3_-hole | Fe-Co bridge | -0.01 | 0.98 | **2.53** | 1.42 |

In our computation modeling, Sv means that FePS_3_ unit has one S vacancy which is connected to Fe atom, 3Sv means three S vacancy connect to Fe in FePS_3_ or (Fe, Co)PS_3_ unit. PSv means we introduced one P and S vacancy into (Fe, Co)PS_3_ unit, and hole means we fabricated hole defects in (Fe, Co)PS_3_ unit.

**Table** s2. Relative contents of surface elements for (Fe_0.53_Co_0.46_)PS_3_

| **Sample** | **Surface composition(at%)** | |
| --- | --- | --- |
|  | Co^2+^/Co^3+^ | Fe^2+^/Fe^3+^ |
| (Fe_0.53_Co_0.46_)PS_3_ | 51.70/48.30 | 39.76/60.24 |

**Table** s3a. The fitting results of the charge transfer resistance (*R*_ct_) and the solution resistance (*R*_s_) for (Fe,Co)PS_3_ catalysts.

|  | FePS_3_ | (Fe_0.66_Co_0.34_)PS_3_ | (Fe_0.59_Co_0.41_)PS_3_ | (Fe_0.53_Co_0.46_)PS_3_ |
| --- | --- | --- | --- | --- |
| *R*_ct_(Ω) | 5048.2 | 980.5 | 493.4 | 65.6 |
| *R*_s_(Ω) | 9.5 | 16.8 | 9.7 | 9.5 |

**Table** s3b. Comparison table for OER performance of (Fe_0.53_Co_0.46_)PS_3_ nanomaterial with the previously reported electrocatalyst.

| Catalyst | Overpotential(mV)  @10mA/cm^2^ | Tafel slope(mV/dec) | Stability(h) | Reference |
| --- | --- | --- | --- | --- |
| **(Fe_0.53_Co_0.46_)PS_3_** | 289 | 58.3 | **138** | **This work** |
| Co_0.45_Fe_0.55_PS_3_ | 310 | 62 | 20 | ^[5]^ |
| FePS_3_ after ball milling | 390 | 58.0 | 12 | ^[6]^ |
| Ni-FePS_3_ NSs/C | 287 | 41.1 | 48 | ^[7]^ |
| Co-FePS_3_ NSs/C | 320 | 52.8 |  | ^[7]^ |
| CoFe-LDH/MXene | 319 | 50 | 10 | ^[8]^ |
| CoPS_3_@DNC | 297 | 51.8 | 20 | ^[9]^ |
| CoFe LDH/Co_3_O_4_(6:4) | 290 | 77 | 10 | ^[10]^ |
| Co-BTC/CC | 370 | 89.1 | 5 | ^[11]^ |
| L-CoOOH | 330 | 63.2 | 10 | ^[12]^ |
| NiPS_3_@G-1:1 | 294 | 42.6 | 30 | ^[13]^ |
| NiFe-NS | 302 | 40 | 13 | ^[14]^ |
| E-CoFe LDHs | 302 | 41 | 10 | ^[15]^ |
| Co_0.5_Fe_0.5_-LDH | 270 | 64.6 | 10 | ^[16]^ |
| Co_2_Ni-MOF@MX-1:1 | 265 | 51.7 | 25 | ^[17]^ |
| CdP_2_-CDs-CoP | 285 | 81.77 | 50 | ^[18]^ |
| La_0.8_Sr_0.2_Co_0.8_Fe_0.2_O | 248 | 51 | 20 | ^[19]^ |
| PO-CoFe-OH | 365 | 121 | 50 | ^[20]^ |
| CoFe-LDHs | 310 | 59 | 12 | ^[21]^ |
| (Ni,Fe)P(S,Se)_3_ | 210 | 34 | 50 | ^[22]^ |

**Reference**

[1] J. P. Perdew, K. Burke, M. Ernzerhof, *Phys. Rev. Lett.* **1996**, *77*, 3865.

[2] G. Kresse, J. Hafner, *Phys. Rev. B* **1994**, *49*, 14251.

[3] H. J. Monkhorst, J. D. Pack, *Phys. Rev. B* **1976**, *13*, 5188.

[4] S. Nosé, *J. Chem. Phys.* **1984**, *81*, 511.

[5] C. Huang, H. Lin, C. Chiang, H. Chen, T. Liu, D. Vishnu S. K, J. Chiou, R. Sankar, H. Tsai, W. Pong, C. Chen, *Adv. Funct. Mater.* **2023**, *33*, 2305792.

[6] W. Zhu, W. Gan, Z. Muhammad, C. Wang, C. Wu, H. Liu, D. Liu, K. Zhang, Q. He, H. Jiang, X. Zheng, Z. Sun, S. Chen, L. Song, *Chem. Commun.* **2018**, *54*, 4481.

[7] C. Tang, D. He, N. Zhang, X. Song, S. Jia, Z. Ke, J. Liu, J. Wang, C. Jiang, Z. Wang, X. Huang, X. Xiao, *Energy Environ. Mater.* **2022**, *5*, 899.

[8] C. Hao, Y. Wu, Y. An, B. Cui, J. Lin, X. Li, D. Wang, M. Jiang, Z. Cheng, S. Hu, *Mater. Today Energy* **2019**, *12*, 453.

[9] P. Liu, Y. Pu, *Int. J. Hydrogen Energy* **2022**, *47*, 197.

[10] P. Ma, H. Yang, Y. Luo, Y. Liu, Y. Zhu, S. Luo, Y. Hu, Z. Zhao, J. Ma, *ChemSusChem* **2019**, *12*, 4442.

[11] S. Naik Shreyanka, J. Theerthagiri, S. J. Lee, Y. Yu, M. Y. Choi, *Chem. Eng. J* **2022**, *446*, 137045.

[12] C. Meng, M. Lin, X. Sun, X. Chen, X. Chen, X. Du, Y. Zhou, *Chem. Commun.* **2019**, *55*, 2904.

[13] S. Xue, L. Chen, Z. Liu, H.-M. Cheng, W. Ren, *ACS Nano* **2018**, *12*, 5297.

[14] F. Song, *Nat. Commun.* **2014**.

[15] P. Zhou, Y. Wang, C. Xie, C. Chen, H. Liu, R. Chen, J. Huo, S. Wang, *Chem. Commun.* **2017**, *53*, 11778.

[16] S. Shankar Naik, J. Theerthagiri, F. S. Nogueira, S. J. Lee, A. Min, G.-A. Kim, G. Maia, L. M. C. Pinto, M. Y. Choi, *ACS Catal.* **2023**, *13*, 1477.

[17] P. Tan, R. Gao, Y. Zhang, N. Han, Y. Jiang, M. Xu, S.-J. Bao, X. Zhang, *J. Colloid Interface Sci.* **2023**, *630*, 363.

[18] Y. Bai, L. Zhang, Q. Li, Y. Wu, Y. Wang, M. Xu, S. Bao, *ACS Sustainable Chem. Eng.* **2021**, *9*, 1297.

[19] C. Zhao, N. Li, R. Zhang, Z. Zhu, J. Lin, K. Zhang, C. Zhao, *ACS Appl. Mater. Interfaces* **2019**, *11*, 47858.

[20] D. Zhong, T. Li, D. Wang, L. Li, J. Wang, G. Hao, G. Liu, Q. Zhao, J. Li, *Nano Res.* **2022**, *15*, 162.

[21] P. Li, M. Wang, X. Duan, L. Zheng, X. Cheng, Y. Zhang, Y. Kuang, Y. Li, Q. Ma, Z. Feng, W. Liu, X. Sun, *Nat Commun* **2019**, *10*, 1711.

[22] W. Li, C. Li, H. Dong, X. Zhang, J. Liu, M. Song, G. Wang, L. Zhao, H. Sheng, B. Chen, H. Zhang, *Angew Chem Int Ed* **2023**, *62*, e202214570.
